# Review of social networks of professionals in healthcare settings—where are we and what else is needed?

**DOI:** 10.1186/s12992-021-00772-7

**Published:** 2021-12-04

**Authors:** Huajie Hu, Yu Yang, Chi Zhang, Cong Huang, Xiaodong Guan, Luwen Shi

**Affiliations:** 1 Department of Pharmacy Administration and Clinical Pharmacy, School of Pharmaceutical Sciences, Peking University, 100191 Beijing, China; 2 Institute of Medical Information/Medical Library, Chinese Academy of Medical Sciences & Peking Union Medical College, Beijing, China; 3 International Research Center for Medicinal Administration, Peking University, Beijing, China

**Keywords:** Health care provider, Social network analysis, Professional network, Umbrella review

## Abstract

**Background:**

Social Network Analysis (SNA) demonstrates great potential in exploring health professional relationships and improving care delivery, but there is no comprehensive overview of its utilization in healthcare settings. This review aims to provide an overview of the current state of knowledge regarding the use of SNA in understanding health professional relationships in different countries.

**Methods:**

We conducted an umbrella review by searching eight academic databases and grey literature up to April 30, 2021, enhanced by citation searches. We completed study selection, data extraction and quality assessment using predetermined criteria. The information abstracted from the reviews was synthesized quantitatively, qualitatively and narratively.

**Results:**

Thirteen reviews were included in this review, yielding 330 empirical studies. The degree of overlaps of empirical studies across included reviews was low (4.3 %), indicating a high diversity of included reviews and the necessity of this umbrella review. Evidence from low- and middle-income countries (LMIC), particularly Asian countries, was limited. The earliest review was published in 2010 and the latest in 2019. Six reviews focused on the construction or description of professional networks and seven reviews reported factors or influences of professional networks. We synthesized existing literature on social networks of health care professionals in the light of (i) theoretical frameworks, (ii) study design and data collection, (iii) network nodes, measures and analysis, and (iv) factors of professional networks and related outcomes. From the perspective of methodology, evidence lies mainly in cross-sectional study design and electronic data, especially administrative data showing “patient-sharing” relationships, which has become the dominant data collection method. The results about the impact of health professional networks on health-related consequences were often contradicting and not truly comparable.

**Conclusions:**

Methodological limitations, inconsistent findings, and lack of evidence from LMIC imply an urgent need for further investigations. The potential for broader utilization of SNA among providers remains largely untapped and the findings of this review may contain important value for building optimal healthcare delivery networks.

**PROSPERO registration number:**

The protocol was published and registered with PROSPERO, the International Prospective Register of Systematic Reviews (CRD42020205996).

**Supplementary Information:**

The online version contains supplementary material available at 10.1186/s12992-021-00772-7.

## Background

The wide application of Social Network Analysis (SNA), especially when used with contact tracing techniques during the control of COVID-19 outbreaks, has attracted considerable public attention [1, 2]. SNA is a research paradigm studying relationships between and among actors (i.e., individuals, organizations or entities) within an interconnected group, and investigating how the patterns of connections impact outcomes of interest [3, 4]. Apart from its utilization in studying the dynamics of the spread of infectious diseases [5] and public health interventions [6], SNA is also increasingly applied to the study of health professional relationships. By exploring professional networks among healthcare providers, SNA has the potential to augment our understanding of uptake of research findings, promote systematic diffusion of evidence-based treatments, influence provider practice, facilitate effective and efficient clinical decision-making, and subsequently improve health-related outcomes.

Previous systematic literature reviews of provider networks differed in their focuses, participants and screening period. For example, Cunningham et al. and Bae et al. studied networks among health professionals in general [7, 8], while Benton et al. studied networks only among nurses. [9]. The frequency of repeated occurrences of primary studies across systematic reviews was also low, indicating a significant inconsistency. Moreover, though the use of SNA in public health reaches a momentum since the early 2000 s, limited evidence supported that network-based intervention realized the potential of SNA. To date, there is limited understanding of how provider networks are formulated, what factors affect network properties, and what network structures are optimal for health outcomes.

To narrow the gaps heretofore mentioned, it is timely to assess the current state of knowledge about the use of SNA in health professional relationships. Given the time and resources constraints, an updated and directed review of hundreds of empirical studies is unlikely to represent an optimal approach. Umbrella review, one of the most common types of reviews [10], specifically refers to a review “compiling evidence from multiple reviews into one accessible and usable document” which focuses on broad condition or problems [11]. Each umbrella review aims to answer what is known and what remains unknown, and provide recommendations for practice and future research. Thus, umbrella review serves as an appropriate and efficient tool for our research question. We aimed to provide an overview of SNA application in health professional relationships in different countries. The review protocol was published and the review was reported according to the Preferred Reporting Items for Systematic Reviews and Meta-Analyses (PRISMA) [12]. This review thus provides evidence and insights for researchers, administrators and policymakers to design tailored behavior-change interventions and clarifies priorities for further research.

The specific research questions developed for this umbrella review were as follows:

1. How have researchers analyzed the social networks of health care professionals in the light of (i) theoretical frameworks, (ii) study design and data collection, and (iii) network nodes, measures and analysis.
2. What factors (i.e., antecedents) influence the formulation and the functioning of interactions among health professionals? How do the patterns of provider networks affect their related outcomes?
3. What is the untapped potential that may inform future research? What are the pitfalls when exploring provider networks?

## Methods

### Literature searches

Articles and reviews were identified through searches in eight academic databases (Pubmed, Embase, Scopus, ProQuest, Web of Science Core Collection, ScienceDirect, SAGE, Wiley Online Library) and grey literature (Google Scholar) from database inception until Aug 31, 2020, and then updated our search on April 30, 2021. Literature search was combined with an extensive manual search of reference lists and related citations. In this review, we operationally defined “health care professionals” as physicians, physician’s assistants, pharmacists, clinical officers, nurses, and others who provide health-related services to patients in formal medical settings. The search concept for SNA was adapted from Chambers et al.’s and Sabot et al.’s systematic review of SNA [13, 14]. We used a snowballing method to identify the list of keywords and to develop the search strategies [4]. The full search query was provided in appendix 1.

### Study selection

The articles were included if they performed a systematic review or systematic literature review on SNA regarding healthcare providers. We focused on provider-to-provider formal or informal professional communication, such as advice-seeking or giving, or discussion about virtual or actual work situations or patients. We excluded: (1) studies conducting SNA of personal relationships such as friendship networks of healthcare workers unless they also captured communication related to patient; (2) studies exploring patient-to-patient networks or patient-to-provider networks (e.g., reviews focusing on the applications of SNA to studying obesity and health behavior [15, 16]) as these studies were not designed to generate insight and thus methods to assess professional communication among providers; (3) studies where networks did not involve healthcare professionals; (4) studies that failed to meet the inclusion criteria; (5) non-English language articles; (6) the publication’s full text cannot be obtained by the review team.

### Data extraction and quality assessment

After downloading articles into Endnote X9.3.1 and removing duplicates, two reviewers (HH and CZ) independently reviewed articles titles and abstracts. Full texts were included if both reviewers identified the articles as relevant. Any discrepancies were resolved by consensus and arbitration by a panel of investigators within the review team (YY, CH, XG, and LS). For the included reviews, we reviewed the main reports and supplementary materials. Summary data from these studies were extracted by three reviewers (YY, HH and CH) using a predefined standardized form to compile a tabular presentation of the following information: title, review purpose or research questions, search database, the total number of empirical studies assessed, range of years reviewed, countries where studies were undertaken, theoretical frameworks of empirical studies, study design and data collection, types of professional networks, network properties, software tools used for SNA, data synthesis and statistical analysis, attributes and consequences. All authors verified the completeness and accuracy of the documentation. We used the AMSTAR (A Measurement Tool used to Assess systematic Reviews) checklist from Shea et al. and Wegewitz et al. [17, 18], a widely used assessment tool, to evaluate the quality of the reviews (appendix 2).

### Synthesis and analysis

After extracting relevant data and assessing study quality, the information abstracted from the reviews was synthesized quantitatively, qualitatively and narratively. To assess reoccurrence of empirical studies across the reviews, citation matrices were generated. “Corrected Covered Area” (CCA) was then calculated (appendix 3), with CCA=0–5 indicating a slight overlap, CCA=6–10 a moderate, CCA=11–15 a high, and CCA > 15 a very high overlap [19]. We then performed a scientometric analysis for empirical studies after removing duplicates. Descriptive statistics (e.g., percentages, frequency counts) were calculated to provide an overview of the literature’s breadth. Summary tables were constructed to simplify data into manageable frameworks and determine common themes.

## Results

### Inclusion/exclusion algorithm and quality assessment

**Fig. 1:**
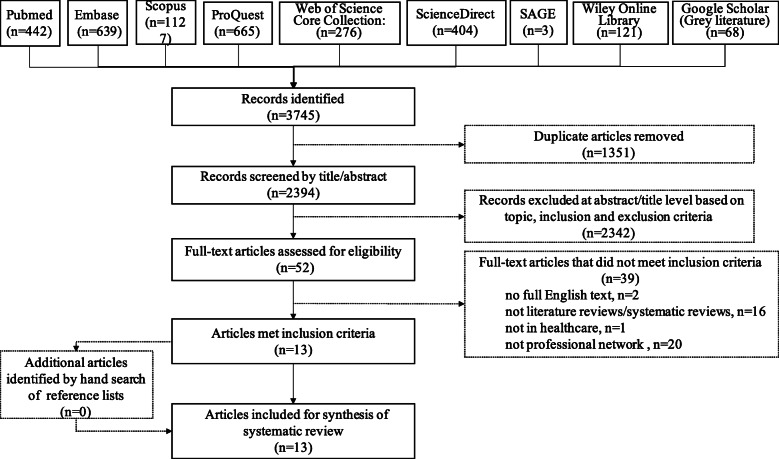
PRISMA (Preferred Reporting Items for Systematic Reviews and Meta-Analyses) flow diagram

After eliminating duplicates, reviewing article titles and abstracts and assessing full-text, thirteen reviews met the selection criteria. The results of article sifting process were presented visually as a PRISMA flow diagram in Fig. 1 and appendix 12.

The 13 full articles was assessed using the modified AMSTAR checklist and was each assigned a quality category of high, moderate, or low. Seven reviews achieved moderate quality whilst the remaining reviews attained high (*N*=5) or low (*N*=1) quality scores (Table 1). The only article rated low quality was incorporated as this article nonetheless offered valuable insights into the use of SNA in healthcare setting [20]. Appendix 4 exhibited the detail of quality evaluation of all reviews.

**Table 1:** Quality assessment results using modified AMSTAR checklist.

No.	Review	State of articles quality
1	Glegg et al. (2019)	MODERATE
2	DuGoff et al. (2018)	MODERATE
3	Brunson et al. (2018)	MODERATE
4	Sabot et al. (2017)	HIGH
5	Poghosyan et al. (2016)	HIGH
6	Mitchell, et al. (2016)	MODERATE
7	Bae et al. (2015)	MODERATE
8	Benton et al. (2015)	HIGH
9	Tasselli et al. (2014)	MODERATE
10	Cunningham et al. (2012)	HIGH
11	Chambers et al. (2012)	HIGH
12	Dunn et al. (2011)	LOW
13	Braithwaite et al. (2010)	MODERATE

*Notes*

Quality Rating Criteria Using AMSTAR Score: low, 0-4; moderate, 5-8; High, 9-11

### Overview of the reviews included

A total of 13 reviews were included in our review (Table 2) – reporting on 330 empirical studies. From the perspective of topic, six methodology-oriented reviews focused on the construction or description of professional networks [9, 21–25], investigating network formation and types; seven result-focused reviews reported factors or influence of professional networks [3, 7, 8, 13, 14, 26, 27], exploring how network properties and structures affected clinician practice and patient outcomes. With respect to the place of conduct, most empirical studies were undertaken in the United States while the remaining studies were conducted in various high-income countries including the European Union countries, Australia and Canada. Evidence from low- and middle-income countries (LMIC) and/or Asian countries was limited. The earliest review was published in 2010 and the latest in 2019.

**Table 2:** Overview of the Reviews Included

Review	Topic	No. of studies	Top three countries reviewed, N (%)	Data collection			Study design				No. of participants (range)
				**primary data**	**Secondary data**	**primary & secondary data**	**experimental design**	**cross-sectional**	**cohort**	**Longitudinal**	
1. Glegg et al. (2019)	Networks and knowledge translation	27 *	USA, 8(29.6 %); Italy, 8(29.6 %); Canada, 4(14.8 %)	19	2	0	0	19	0	2	13~784
2. DuGoff et al. (2018)	Patient-sharing network using administrative data	49	USA, 37(75.5 %); Australia, 6(12.2 %); Italy, 3(6.1 %)	0	49	0	0	39	6	4	N/A
3. Brunson et al. (2018)	Applications of network analysis to health care data	189 †	N/A	0	189	0	N/A	N/A	N/A	N/A	N/A
4. Sabot et al. (2017)	Professional advice and performance among provider	6	USA, 5(83.3 %); Australia, 1(16.7 %)	5	0	1	1	5	0	0	21~150+
5. Poghosyan et al. (2016)	Health care team networks and their antecedents or consequences	25	USA, 25(100 %)	15	5	5	N/A	N/A	N/A	N/A	25~68,288
6. Mitchell et al. (2016)	Social-professional networks in long-term care settings with people with dementia	4‡	Netherlands, 3(75.0 %); Canada, 1(25.0 %)	4	0	0	0	4	0	0	93~380+
7. Bae et al. (2015)	Social networks and its relationships to care process and patient outcomes	28**	USA, 14(50.0 %); Australia 4(14.3 %); Netherlands, 3(10.7 %)	21	5	2	0	22	0	3	5~61,461
8. Benton et al. (2015)	Thematic analysis of nurse-related social network	43	USA, 21(48.8 %); Canada, 4(9.3 %)	33	9	1	2	32	N/A	5	10~1999
9. Tasselli et al. (2014)	Antecedents of health care professionals’ social networks and their consequences	85	USA, 36(42.4 %); UK, 12(14.1 %); Italy, 10(11.8 %)	78	5	2	N/A	N/A	N/A	N/A	N/A
10. Cunningham et al. (2012)	Professional networks to improve quality and safety	26	USA, 13(50 %); Australia, 4(15.4 %); Canada, 3(11.5 %)	20	2	4	N/A	5	N/A	N/A	21~520
11. Chambers et al. (2012)	SNA to support the implementation of change	52††	USA, 25(48.1 %); Netherlands, 6(11.5 %)	48	2	2	1	47	N/A	N/A	N/A
12. Dunn et al. (2011)	Validating small networks in healthcare organisations	3	USA, 2(66.7 %); Australia, 1(33.3 %)	3	0	0	0	2	0	1	19~31
13. Braithwaite et al. (2010)	Network properties in healthcare	13	UK, 4(30.1 %); USA, 3(23.1 %); Australia, 3(23.1 %)	13	0	0	N/A	N/A	N/A	N/A	9~615

*Notes*:

Primary data for construction of SN: survey, interview, focus group discussions or observation;

Secondary data for construction of SN: document review, document analysis, archival data, examination of secondary survey data, or administrative data (e.g., insurance claims, all-payer datasets —government claims data, private insurance claims data — or electronic medical record);

Primary & secondary data: e.g., linked survey and administrative data;

Experimental design: randomised or non-randomised studies of healthcare interventions

N/A: not applicable or not stated

*: only 21 studies’ data sets were described;

†: 189 distinct studies, presented in 200 publications (138 journal articles, 52 conference presentations (papers and extended abstracts), 9 book sections, and 1 electronic preprint);

‡: another five studies focused on residents and were not included in this umbrella review;

**: 28 unique studies were published in 29 articles. Another 3 study design types were: mixed methods (*n *= 2) and qualitative (*n* = 1);

††: 52 completed studies were reported in 62 publications. Another 4 study design types were not stated

Annual publication trends in the scientometric summary of empirical studies showed a sign of emerging interest in provider networks (appendix 5). The original research in SNA of health professional networks could be traced back to 1957 and its popularity has shown momentum since the early 2000 s. The peak time of publication was between 2010 and 2016. The empirical studies identified had several “home journals” (i.e., publication central hubs), such as Social Science and Medicine, BMC Health Services Research, Journal of the American Medical Informatics Association, Medical Care and PLoS One (appendix 6). The CCA was 0.043 in included reviews (4.3 % overlap). This means that most empirical studies were only cited in a single review and not in multiple reviews (appendix 3). The citation matrix for reviews was presented in appendix 11 and any overlap of studies across the included reviews was indicated in the table.

### Utilization and potential of SNA regarding healthcare providers

The included reviews demonstrate a wide application of SNA to study health professional networks. Most health services studies have explored health care delivery using traditional social science methodologies that focus on individual-level factors and fail to capture relational structures within groups [3, 26]. Conversely, SNA focuses on dynamic relationships among group members. It provides a network-level perspective to investigate how professionals engage in social interactions at work and how professional networks affect outcomes of importance at both individual- and group- levels [3]. In practice, the term “network” is often interchangeable with “collaboration” in that it captures the performance and interactions of teams and organizations [8]. Among all reviews included in this paper, 11 proved SNA’s validity in unveiling and mapping channels of communication and information flow, collaboration, and disconnects among health care providers [8, 9, 13, 14, 21–27]. Understanding and harnessing the power of existing professional networks could facilitate the quality of healthcare delivery and enhance patients’ health outcomes [14]. Moreover, provider’s characteristics in the relationships with others has been identified as a critical factor to defining the opportunities and constraints a provider may encounter in providing services. SNA is used to evaluate key network characteristics, structures, and positions of relevance to knowledge translation and diffusion of innovation [3], and to investigate transmission of resources [7], and leverage relational structures to accelerate practice behavior change [26]. Due to the advantages identified, health system reform in many countries has looked to network governance [23].

Indeed, all included reviews suggested that SNA showed considerable potential in creating cohesive and collaborative professional networks [9, 27]. It was deemed promising in coordinating and enhancing the quality and safety of care in four ways. Firstly, SNA could be used to identify key providers within the network as a tipping point for shift diffusion of information and innovations [3, 7, 26]. These key providers with influence could act as connectors to transmit information, inducing better team performance and quality of care. Secondly, the identification of peripheral individuals or isolated subgroups in the network, who might negatively affect team productivity and performance, helps managers reallocate work schedules and build a bridge for them to promote communication. [26]. Thirdly, administrators could use SNA results to construct or redesign provider networks with the optimal professional mix, ideal composition, high-performing network structure and appropriate network size to achieve optimal outcomes [9, 21, 25, 26]. Fourthly, policymakers could develop tailored interventions for individuals based on their network structure or attributes to promote collaboration, knowledge translation, and ultimately health outcomes [3, 13, 14, 26]. Furthermore, at an advantage in monitoring network strengths and gaps or barriers, SNA can be paired with the conventional educational program or behavioral outcomes approach to realize network structures’ full potential [3]. More details about the utilization and potential of SNA regarding healthcare providers were presented in appendix 13.

### Theoretical frameworks

The typical theoretical frameworks in health professional networks, for a more comprehensive understanding, could be divided into two categories: (1) social network theory or SNA paradigm; (2) non-SNA-specific theory derived from other fields (e.g., sociology, psychology, epidemiology, and economics).

In most articles, SNA was employed to conduct exploratory analysis and to identify and interpret network properties. Studies were most frequently built on ***Mark Granovetter’s conception of weak ties*** hypothesizing that weak ties (i.e., relationships that are occasional and incidental) could accelerate the dissemination of information, bridge networks, and increase actors’ mobility [28, 29]. Some studies were based on the conception of ***structural holes*** (i.e., concepts that describe the absence or rarity of connections between cohesive subgroups in a network) and generally examined the association between structural holes and the establishment of brokers that bridged the gap within the network [30]. Other studies evaluated network dynamics related to ***embeddedness*** (i.e., a concept that describes how an individual’s directly connected peers relate to each other), ***tie homophily*** (i.e., the tendency of similar people to have a relationship) [31]. For other less used SNA paradigms, refer to Table 3.

**Table 3:** Summary of theoretical or conceptual frameworks in included reviews

Review	Theoretical or conceptual frameworks (No. of studies)
1. Glegg et al. (2019)	(1) theory drawn from the fields of sociology and psychology: Diffusion of innovation (*n*=7), social contagion (*n*=4), and social influence (*n*=3) were most commonly applied. (2) SNA-specific theory (n=6): weak ties, structural holes, cohesion, or tie homophily (3) SNA paradigm without reference to a specific theory (*n*=7)
2. DuGoff et al. (2018)	(1) networks reflect aspects of collaboration, continuity, and care coordination (2) Mark Granovetter’s strength of weak ties (3) other studies examined how networks influence the adoption of medical technology into clinical practice (diffusion of innovation). (4) patient-sharing relationships serve as a vector for the spread of infectious diseases.
3. Brunson et al. (2018)	care coordination (*n*=16), collaboration and competition (*n*=10), collaborative practice (*n*=11), decision support (*n*=6), organizational effectiveness (*n*=11), social capital and social influence(*n*=7), health surveillance (*n*=5), inappropriate access (*n*=4)
4. Sabot et al. (2017)	(1) diffusion of innovations (2) knowledge translation and transfer.
5. Poghosyan et al. (2016)	(1) professional networks: advice and consultation regarding patient care, exchange of information and knowledge, adaptation of prescriptions and treatments, patient sharing or referral, and research and professional development. (2) personal networks: mainly characterized by interactions regarding friendship and emotional support (e.g. creating leisure ties, interacting socially).
6. Mitchell et al. (2016)	health professionals and social context, social support, information exchange, social influence, service provision/organisation
7. Bae et al. (2015)	(1) identification and interpretation of clusters: validity of weak ties/structural holes theories (*n* = 10) and study network member embeddedness (*n*=2); (2) social influence effects: theories of information exchange (*n* = 21) and resource exchange (*n* = 1) and exploring the notion of trust between network members (*n* = 2); (3) centrality metrics interpretation: theories of social capital (*n* = 2), social support (*n* = 3), and studying prestige (*n* = 2); (4) network formation principles (*n* = 5): studying in-network reciprocity, proximity, transitivity, homophily, and small-worldness theories.
8. Benton et al. (2015)	Thematic analysis: network architecture, roles that individuals played, communication structures, power relationships, opinion leaders, differing advice-seeking patterns
9. Tasselli et al. (2014)	homophily theory; knowledge transfer, diffusion of innovation in organizations, and organizational performance; interpersonal networks in organizations as structures of constraint and opportunity negotiated and reinforced through professionals’ interactions
10. Cunningham et al. (2012)	(1) structural relationships within and between organisations (*n*=6); (2) health professionals and social context (*n*=13, including six on work climate); (3) structure of quality collaboratives and healthcare partnerships(*n*=4); (3) structure in knowledge sharing networks(*n*=4)
11. Chambers et al. (2012)	social networks in relation to service provision and organisation (*n*=19), the role of social networks in the context of behaviour change (*n*=22 studies, including diffusion of innovations, opinion leaders and other aspects of social influence), decision-making(*n*=1), interpersonal relations(*n*=1), information sharing behaviour (*n*=1), social support(*n*=3).
12. Dunn et al. (2011)	professional networks: team communication (*n*=2); structure of quality collaboratives and healthcare partnerships (*n*=1)
13. Braithwaite et al. (2010)	new public management theory; culture theory; change, particularly structural change; organizational change theory; social network theory; strategic leadership process theory; organizational culture and sub-culture theory; nursing socialization theory; structuration theory; social identity theory; learning theory within complex adaptive systems; decision theory in real world settings; acquisition theory; boundary roles and boundary- spanning theory; social influence theory;

*Notes*:

Summations and proportions of empirical studies in included reviews presented might not sum to 100 % in cases where articles did not present related information or where the categories of characteristics were not mutually exclusive

Among studies that obtained theoretical frameworks from other disciplines, ***diffusion of innovation*** was the theory most commonly referred to when analyzing how information spread varied with different network positions and other attributes [32, 33]. Health policy and health systems research also employed the conceptions of “knowledge translation and transfer” to capture the diffusion of innovation in terms of knowledge sharing [34–36]. Another commonly used concept was ***care coordination***, which encompassed collaboration, continuity, collaborative practice, healthcare partnerships, professional advice seeking and information exchange. Studies employing the conception of care coordination suggested that network reflected aspects of coordination and hypothesized that strongly connected providers were associated with high-quality health care and the dissemination of integrated and organized practices [37–39]. Another theory frequently referred to was ***social contagion/influence theory*** (i.e., the performance of peers to whom the provider was closely connected). This perspective explored how the structure and other properties of networks facilitated or hampered the norms that impacted provider attitudes and behaviors [40]. A growing body of studies focused on the role of peers (e.g., opinion leaders, connectors and bridges or brokers) in the formulation of social influence [32, 41–43]. Based on the information-transferring nature of professional networks, the definition of these frameworks above often overlapped. Each of 13 reviews classified frameworks by subtly different standards, and other theories were presented in Table 3.

### Study design and data collection of empirical studies

The majority of empirical studies included in 13 reviews were cross-sectional, observational study with only a few longitudinal(Table 2), though cross-sectional study design is likely to limit the generalizability of study findings as it risks overlooking the network dynamics within groups. Only four empirical studies involved in the reviews used an experimental or quasi-experimental design. Among these studies, Lindberg et al.’s study was thought to preclude a network-based intervention because they did not quantify network data to design the intervention [44]. The other three using the results of SNA as part of an intervention to improve care delivery had some design limitations, including non-random assignment and remarkable between-group differences at baseline [45–47].

The vast majority of studies on professional networks in healthcare settings relied on primary data (e.g., survey, interview, focus group discussions, or observation), which was consistent with the patterns in SNA-related research conduct(Table 2) [48]. Compared with secondary data (e.g., document review, archival data, administrative data), the use of original surveys and interviews to quantify networks was more time- and resource-consuming, which imposed constraints to network modeling. For networks of small size, the metrics (e.g., clustering, centralization, and hierarchy metrics) used during analysis were sensitive to changes in the number of people and the density of observed relationships. This feature made comparisons amongst networks of different sizes and generalizations of the consequences in small networks difficult [24]. With the improvement of electronic information systems, secondary data collection enabled researchers to obtain large-scale social networks in greater depth and variability after the year 2000 (Table 2). Recently, administrative data using “patient-sharing” relationships became the dominant method of secondary SNA data collection. In studies exploring administrative data like this, pairs of physicians or other providers were considered connected if they both delivered care to the same patient.

### Network nodes, measures and analysis

The empirical studies included in the 13 reviews demonstrated a broad range of actors or nodes from micro-level interpersonal networks to meso-structure subgroups to macro-level institutional exchange networks. At the individual level, more than half of empirical studies examined physician networks. Some studies focused on nurse-related networks while only a few explored other provider networks or interdisciplinary teams (Table 4). Only three reviews reported health settings where participants were surveyed and over half of the studies were conducted in hospitals with the remaining studies in outpatient clinics, long-term care or multi-disciplinary research institutes (appendix 10).

**Table 4:** Network nodes in included reviews

Review	No. of studies reviewed	No. of studies of different network nodes			
		Physician networks	Nurse networks	Other providers/ Interprofessional networks	Inter-organizational networks
1. Glegg et al. (2019)	27*	11	1	9	0
2. DuGoff et al. (2018)	49	----------------------------------------36----------------------------------------			13
3. Brunson et al. (2018)	189†	33	0	47	50
4. Sabot et al. (2017)	6	0	2	4	0
5. Poghosyan et al. (2016)	25	12	4	9	0
6. Mitchell et al. (2016)	4	0	3	1	0
7. Bae et al. (2015)	28	10	6	12‡	0
8. Benton et al. (2015)	43	0	43	0	0
9. Tasselli et al. (2014)	85	N/A	N/A	N/A	N/A
10. Cunningham et al. (2012)	26**	2	3	19	0
11. Chambers et al. (2012)	52	19	9	24††	0
12. Dunn et al. (2011)	3	0	0	3	0
13. Braithwaite et al. (2010)	13	2	1	10	0

*Notes*:

N/A: not applicable or not stated

* only 21 studies’ data sets were described;

†: clinical co-occurrence networks (*n*=59) were not explicit professional network so not presented here;

‡: 10 studies included multidisciplinary teams (interprofessional clinicians) and two studies focused uniquely on administrators or infection control specialists;

**: 24 of the 26 studies were directed at health professionals. Other providers/ Interprofessional networks (*n*=19) : multidisciplinary, 7; Mental health professionals, 5; Health service managers or administrative staff, 4; Varied health professionals, 2; Dementia care professionals,1;

††: teams or mixed groups of health professionals (17 studies); other health professionals including administrators, emergency planners and policy makers (seven studies)

Measuring network properties to capture the characteristics of participants is a distinctive characteristic of SNA. Network properties are calculated to demonstrate how strong or far two nodes were connected, how central the node is located at, how many connections the network has, how dense the network is, the hierarchical structure of the network, and so forth [7]. These properties help determine how well providers coordinated with each other and how properly the network functiones [9]. Nearly all reviews grouped network properties into different categories at individual-, dyad- and triad-, network-, organization- and patient-level (Table 5). There was a surprising variability across the empirical studies regarding the network measures, with 7 of 13 reviews presenting more than ten network metrics, and up to 180 social network measures were used to describe relationship data in one review [21]. At the individual level, the most commonly used network indicators were related to the “connectedness of nodes” (e.g., in-degree, out-degree), “location of individuals” (e.g., betweenness centrality, closeness centrality) and “roles that actors played” (e.g., broker of information, leadership position) [9]. Though network visualizations can optimize SNA results presentation and dissemination, only three reviews exhibited visualizations to depict network property configurations and fewer than half of the empirical studies mapped information flow [3, 8, 14].

**Table 5:** Social network measures and analysis

Review	Network measures (No. of empirical studies)	Methodological framework to test hypotheses (No. of empirical studies)
1. Glegg et al. (2019)	(1) network properties (*n*=8): 28 structural properties, with degree centrality, tie characteristics (e.g., homophily, reciprocity), and whole network density being most frequent (2) network visualizations (*n*=13) (3) conventional descriptive statistics (*n*=2): e.g., frequency counts, proportions.	(1) regression (*n*=14): ordinary least squares, ordinal logistic regression, multi-level modeling, P2 logistic regression, linear regression (2) paired t tests or Wilcoxon ranks (*n*=3) (3) Chi-square test (*n*=2) (4) exponential random graph models (*n*=3); quadratic assignment procedure (5) factor analysis: *n*=1
2. DuGoff et al. (2018)	(1) provider-level: centrality, degree, density (2) dyad- and triad-level: Assortativity, distance, edge, Jaccard similarity, reciprocity, recurrence, transitivity (3) patient-level: care density, team size, provider constellation	(1) a range of different statistical approaches from correlation coefficients to multilevel regression modelling examine the association between network characteristics and aspects of health care utilization (2) Girvan-Newman algorithm (*n*=6) and studies used the Blondel model (*n*=2) to identify clusters of providers (3) Exponential-family Random Graph Models (*n*=3); Multiple Membership Multiple Classification model(*n*=1)
3. Brunson et al. (2018)	motifs, neighbourhood, meso-structure, distance effects	regression (*n*=27) exponential random graph model (*n*=6) rule mining (*n*=4)
4. Sabot et al. (2017)	clustering coefficient, component count strong, component count weak, density, diffusion, fragmentation, hierarchy, isolates, centrality, simmelian ties, number of triads, and number of cliques, degree, connectivity, inclusion, reach, and centralization, reciprocity, tie strength	(1) correlations (Spearman Rho), Pearson X 2 and Fisher’s exact test, t test, Chi-squared (2) multiple linear regression, generalized linear mixed models (3) qualitative analysis: reflexive observation and contextual analysis, axial coding, themes developed using human factors theory
5. Poghosyan et al. (2016)	(1) individual level: centrality, betweenness centrality, degree centrality (2) team level: centralization, density, hierarchy, cohesion (subgroup property), isolates, clustering, reciprocity	N/A
6. Mitchell et al. (2016)	density, network role, bridging, size and type of tie (i.e., embedded, boundary crossing), density	descriptive analysis using block models, bivariate and multivariate analyses
7. Bae et al. (2015)	(1) actor-level (*n*=18) (2) dyad-level (*n*=7) (3) network-level (*n*=23) (4) organization-level (*n*=6)	group cohesiveness analysis (*n*=18), centrality analysis (*n*=16), regression (*n*=5), monadic or dyadic or network hypotheses (*n*=4), structural equivalence analysis (*n*=2), visual inspection (*n*=3), block model analysis (*n*=2), multidimensional scaling, hierarchical clustering, smallest space analysis, social relations model, correlation analysis
8. Benton et al. (2015)	(1) individual-level, the most frequently reported: in-degree and out‐degree (2) network-level, the most frequently reported: network densities, network centrality	N/A
9.Tasselli et al. (2014)	network density, centrality, and brokerage	N/A
10. Cunningham et al. (2012)	Three levels: actors, the network (or organisation), and inter-network (or inter-organisation) organisation (*n*=8) actors and network(*n*=17, three looked at the actors and team) actors, organisation and external network (*n*=1)	(1) SNA (2) other analysis: sociometric analysis, content analysis, multiple regression (*n*=4), T-tests, survival analysis
11.Chambers et al. (2012)	N/A	N/A
12. Dunn et al. (2011)	(1) indicators of the aggregate properties of networks (2) indicators based on the locations of individuals within networks	social network analysis, qualitative content analysis
13.Braithwaite et al. (2010)	N/A	social science mixed methods

*Notes:* Summations and proportions of empirical studies in included reviews presented might not sum to 100% in cases where articles did not present related information or where the categories of characteristics were not mutually exclusive.

N/A: not applicable or not stated.

With a substantial majority of observational studies using a cross-sectional design, the most commonly observed statistical techniques were ***regression*** and ***bivariate analyses***, both of which examined the association between network properties and aspects of health care utilization [3]. For studies using longitudinal designs, ***stochastic actor-based network models*** (SABM) were applied to examine network change over time [49]. All of these identified traditional analytical approaches required that data satisfied the assumptions of independence, however, dyadic data could not meet these statistical assumptions because of the interdependent nature of network data. Several analytical techniques were therefore designed to account for interdependencies and considered more robust, including ***exponential random graph models*** (ERGM) [30, 50–54], ***quadratic assignment procedure*** (QAP) analysis [55], ***multiple membership multiple classification model*** [56]. ***Girvan-Newman algorithm*** and the ***Blondel model*** were applied to identify ‘communities’ (i.e., clusters of providers who were connected with providers within the group more frequently than with providers outside the group) [57]. For other analysis techniques, refer to Table 5.

### Relationship between antecedents and network features

Researchers have investigated the evolution of networks in healthcare settings. Three factors were identified to have a primary influence on the formation of provider networks: (1) demographic and professional characteristics of providers; (2) environmental and organizational characteristics; (3) characteristics of the patient served (appendix 7).

*Demographic and professional characteristics of providers*. Providers with similar demographics (e.g., age, gender) were more likely to be connected [26]. Similar professional characteristics (e.g., professional affiliations, specialty groups, administrative role or rank, clinical experience, medical school, research orientation) also appeared to be predictive of the network ties between providers. Individuals were more likely to be connected to persons with the same profession (e.g., nurse to nurse, physician to physician) and with membership of the same specialty group (e.g., family practice, surgery, geriatrics, internist) [8, 26]. Boundaries between professional groups might inhibit interprofessional interactions [8, 58, 59].

*Environmental and organizational characteristics* (e.g., occupational distance among members, organizational arrangements, departments) affected providers’ interaction patterns [3]. Providers’ connections were strengthened if the departments where they practiced were geographically close. In addition, organizational arrangements facilitated social interactions between providers as they were interdependent in their common work activities [8, 60].

*Characteristics of the patient served*. Networks were frequently formed among providers who cared for patients with similar age, comorbidities, health status, type of health insurance and racial composition [8, 26]. Providers had a higher likelihood to report a relationship with another provider once they had similar patient populations or shared patients [61]. The size of patient population was considered as another important factor for network density and provider role. In Keating et al.’ study, doctors providing services for a limited number of patients were located in the periphery of the network while physicians with larger patient panels developed denser networks [62].

Whilst most studies reached a point where providers with similar demographic and professional characteristics were more likely to develop network ties, two reviews identified some conflicting findings. Glegg et al.’s review observed inconsistent findings regarding the influence of attitudes towards evidence-based practice, experience, gender, and geographical proximity on tie formation [3]. Poghosyan et al.’s review also identified conflicting reports regarding ties between members of similar demographic characteristics (e.g., similar age, or the same gender) and professional affiliations [26]. Apart from these inconsistent findings across different empirical studies, internal inconsistency was identified in one study, which suggested that male physicians were more likely to have ties with other male physicians while female physicians were less likely to connect with other female physicians [61]. More details were presented in appendix 9.

### Relationship between network feature and health-related consequences

After reviewing the methodologies and theoretical frameworks and conceptions of the reviews, the next question naturally arose: what did these studies find? The following health-related consequences were identified in a relationship with professional network properties: (1) influence on professional behaviors; (2) organizational outcome and performance of coordination; (3) quality of care and patient outcomes; (4) health care utilization and costs (appendix 8).

*Influence on professional behaviors*. The peer-group effects on clinical practice or clinician subgroup membership are predictive of similar professional behavior. Pollack et al. found that a surgeon’s peer group use of imaging studies and brachytherapy influenced hers [21, 63, 64]. Among peers, network location helped determine the size of peer effects [8]. Providers occupying central network positions in social networks tended to be considered as opinion leaders who influenced others’ adoption of new technology, prescriptions, or treatments. However, peer did not have influence on all professional behaviors alike. For example, the use of electronic health records was not associated with physicians’ professional networks [26]. The relationships between adoption and professional advice networks differed across different studies (appendix 9) [27].

*Performance of coordination and organizational outcome* (i.e., efficiency and deficiencies of organization in health care delivery). Several papers found that increasing density, close ties among the interprofessional network, and centrality in the social network enhanced care delivery coordination and efficiency [8, 65, 66]. Among peripheral network members, more external connections were positively associated with collaboration performance [26, 27]. Several other studies investigated whether patient-sharing relationships increased the incidence rate of infectious diseases [21, 67–70].

*Quality of care and patient outcomes*. SNA enables researchers from different professions to explore the association between provider networks and clinical outcomes [7, 71]. Through the clinical work system including teamwork, communication, organizational culture, social climate and organizational arrangements, features of providers’ social networks were found to impact quality of care, which subsequently influences patient outcomes [7, 8]. Interconnected networks implied better outcomes in care delivery compared to networks lacking connections [26]. For example, coordination, as measured by network indicators, was associated with better patient outcomes including fewer inappropriate medications, less emergency department use and fewer hospital readmissions [21].

*Health care utilization and costs*. Studies exhibited statistically significant negative associations between network properties of coordination (e.g., primary care physician centrality, care density, bipartite clustering) and health care utilization and spending measured using the length of stay [21].

The evidence about the impact of health professional network was based on the hypothesis that networks with more connections enjoys better coordination whereas unconnected networks risk greater incidence of communication errors. It is also built on the hypothesis that cohesive and collaborative networks have better organizational outcomes, enhanced quality of care, increased cost-effectiveness of health services, and better managed medical expenditure. However, through testing hypotheses, studies showed inconsistent findings, in part due to different network indicators, analytical approaches and data sources, but even for the most frequently used metrics—centrality and density—the correlation or causal relationship was indiscernible. For centrality, providers who assumed a central role showed a higher propensity to collaborate with others [8, 72], bridged information transmission, facilitated communication and trust, and subsequently improved care delivery. Nonetheless, relying too heavily on the central roles made provider networks vulnerable to negative outcomes [8, 14]. Denser networks generally provided more pathways for information to improve patient outcomes but overly dense networks increased insularity and limit external communication [14].

## Discussion

### Theoretical perspectives: multiple theories from various fields

Various theoretical approaches were used in hypotheses testing or results interpreting, which could split neatly into two groups: those based on social network theory or SNA-specific paradigm and those drawn from the fields of sociology, psychology, epidemiology and economics. These theoretical frameworks was utilized to guide the development of hypotheses and the selection of network attributes [3, 22]. In this way, theories served as building blocks in exploring concordance between the theoretical constructs of interest and the indicators used to detect them. For studies that were not guided by a clear theoretical framework, professional networks were characterized as a “social glue” in provider interaction and theories only played an assistive role in introducing topics or interpretating findings. In these cases, network properties were selected without specific motivation and thus the interpretation of results seemed subjective and needed clearer justification [3, 22]. For example, Kwan TH et al. attempted to provide a reasonable interpretation of the effect of degree and betweenness centrality on the consistency of continued methadone treatment of drug users but admitted that the cause-and-effect relationship could not be confirmed from their results [73, 74].

### Insights about study design and data collection: primarily cross-sectional designs using primary data

While reviewing the methodologies of included articles, we found a low level of technical sophistication in general. Indeed, current studies using SNA were dominated by cross-sectional observational design, in the absence of replication, both over time or with different care teams in the same setting and/or the use of comparisons between similar groups in multiple settings. There were three limitations of this design: (1) studies were conducted in single point-in‐time and consequently, the lack of replication weakened the generalizability of study findings; [9] (2) the timeframe of network constructing and that of the patient outcome being captured were not always temporally aligned; [14] (3) the ability of studies to address causal pathways was limited [14, 75]. To address causal pathways, longitudinal and experimental designs offered an advantage to minimize potential biases. However, related evidence was restricted, which challenged the reliability of cross-sectional findings and constrained the uptake of findings.

Nevertheless, with the popularization of electronic information systems, the use of secondary data, including electronic data and especially administrative data using “patient-sharing”, can regularly monitor providers’ communication patterns to allow for a longitudinal and experimental design; however, DuGoff et al.’s review suggested that the literature on “patient-sharing” employed a diversity of measures and indicated an enduring uncertainty in how to best construct patient-sharing networks [21]. Different studies adopted different statistical approaches and thresholds on the strength of patient-sharing relationships, none of which have become the primary standard [22]. Although a few studies used various techniques to avoid bias or tested the sensitivity of their findings to different thresholds, more validation studies are needed to address stakeholders’ concerns about the validity of SNA in healthcare [22].

### Network nodes and analysis: lack of evidence studying interdisciplinary health care teams and using interdependency-specific analytic statistics

More than half of empirical studies focused on physician networks and most of them centered on intradisciplinary connections. There might be two main reasons. Firstly, providers tended to cluster with those of the same profession [7, 58, 59, 76]. This homophily might cause great difficulty in constructing multidisciplinary collaborations across networks. Secondly, to simplify research conduct, studies often limited participants to a specific profession by setting artificial boundaries, which ignored a few but important interprofessional communications in real-world environments. However, with an increasing shift from profession-centered practice to patient-centered interdisciplinary collaboration, there is a great opportunity to expand the utility of SNA beyond physicians or a single profession in near future.

Given the interdependent nature of network data within the SNA paradigm, several approaches (e.g., ERGM, QAP) were designed to account for interdependencies but none of them have become standard techniques. The traditional analytic statistics, which were designed for data meeting assumptions of independence, still dominated the existing literature. Clustering algorithms to identify subgroup or virtual communities and visualizations to picture communication patterns were another two prevalent hallmarks of SNA, both of which were used less and merited broader utilization and deeper exploration.

### Antecedents and Health-Related Consequences: Inconsistent findings await further investigations

There existed an inconsistency of results though most studies reached consensus about the antecedents of provider networks, which might be partially explained by the variability in study designs and needed future research. Geographic scope and specialties were two common factors impacting the formation of provider networks. Depending on study questions, researchers should carefully and clearly define the geographic and profession boundaries because a subtle difference in exclusion criteria might alter the network structure and its association with clinical outcomes observed by the study. Further research is needed to understand how these factors affected interaction patterns among providers [13, 14, 21].

One lesson learned from existing literature is that an effective network would encourage communication and subsequently improve care delivery. However, the results of different studies were often contradicting and not truly comparable though they were based on the same theoretical construct. Firstly, as topics varied, network members were often heterogenetic among empirical studies, and the SNA metrics and outcomes of interest calculated across studies were often different [14]. Secondly, as mentioned above, the debate about the antecedents and their association with network properties remained. The number and types of included covariates varying widely might explain why studies examining the same measure sometimes disagreed [21]. Thirdly, the construction of the network model is highly dependent on data sources, each of which had its own scope, bias, and format [22]. These reasons also explained why there previously lacked a meta-analysis.

Inconsistent findings inferred that the results should be interpreted with caution. There seemed to be a balance threshold for a network property and it would be elucidated differently once exceeded the threshold. For example, frequent communication indicated by a high degree or density improves coordination, but once exceeded the threshold in communication frequency, the benefits from network collaboration will be counteracted by “information overload”. Additionally, in cases where providers occupied a central position for reasons other than professional communication or advice seeking, the identified significant relationship between centrality and patient outcomes was ambiguous [14].

### Knowledge gaps and future research agenda

Although the rapid development of analytical tools, including software and algorithms, supports the utilization of SNA and generates huge potential in clinical care [9], the state of the science of SNA in healthcare settings stays in a primary phase, compared with its use in the commercial sector [8, 13]. Almost all included reviews identified knowledge gaps and constructed their wish lists for future research (appendix 14). However, after the earliest review found the lack of validation in published healthcare social network analyses in 2012 [13], the latest review in 2019 still observed that most studies were purely descriptive and called for enhanced sophistication in study design (e.g., longitudinal and experimental research) and analysis. The slow pace of research in this field indicates that there must be some problems to narrow the apparent gaps mentioned in included reviews. We discuss potential problems and set a brief agenda for future studies. There are mainly three concerns hindering the utilization of SNA regarding healthcare providers.

First, SNA focuses on the structure of networks and assumes that associations within networks are important [77, 78]. However, previous studies on the SNA of health professionals frequently ignored whether providers perceived the importance of their networks the same way as researchers did. Researchers often define provider relationships from two perspectives: self-defined (i.e., providers decide whether the connection exists and provide the information to the researcher through survey or interview) and other-defined (i.e., researchers infer providers’ relationships from secondary data such as administrative data) [79]. For the former, the relationships were assumed to be similarly important to all providers, even though it might not be true; for the latter, studies assumed increasing strength of association inferred increasing importance. In both cases, researchers’ assumption about the importance of relationships to providers involved in networks was not justified. Given that the relationship’s perceived importance to involved providers likely impacts the magnitude of their preference to interact with each other, further studies should capture what the relationships being studied means to the providers involved. Answering this question may help researchers move beyond the simple hypothesis like more associations, more important and closer relationships, and promote the potential strengths of findings.

Second, almost all previous studies only considered antecedents that occurred within the opportunity structure (i.e., providers’ distributions across categories within a researcher-defined context decide the probability they associate with others in the network) [79], including profession, organization and patient characteristics mentioned above. Scholars tended to neglect providers’ individual preferences (i.e., provider’s inner tendency to select peers who she associates with). Such ignorance might result from the big split between micro- and macro-level provider-network data collection. With the increasing prevalence of the use of electronic administrative data, secondary quantitative data has dominated the existing literature of large-sized provider networks, which limited the depth and length of investigation on a given topic. However, investigating provider’ individual preferences often require qualitative data acquired from an interview or focus group discussions. To narrow the gap, two reviews included in this paper suggested that integrating qualitative methods into quantitative studies could be promising to answer the questions about providers’ individual preferences [3, 14]. The use of mixed-methods SNA is considered an appropriate means to generate an in-depth understanding of the results [78, 80–82].

Third, as an emerging research field, the SNA of health professionals has not developed typical measures. As mentioned in the section of results, up to 180 social network measures were used to describe provider relationships. Even for the most frequently used metrics (e.g., degree, centrality, and density), the variations existed in empirical and theoretical definitions across various studies, which might describe different phenomena in practice. Admittedly, there is no perfect measure. The problem is taking the weakness of measures for granted and overlooking the nuances between measures and researchers’ intended theoretical meaning [79]. The ambiguities in the definition of measures might partially account for the inconsistency in findings and hinder the utilization of SNA in clinical management [8]. To remove the obstacle, on one hand, the research community needs to collaboratively reevaluate the use of network measures, and establish a consensus on the guideline for the utilization of SNA in healthcare settings, including study objectives, perspective, population, time horizon, and so forth [13]. On the other hand, further studies should explicitly disclose the specification and limitations of measures being chosen, capture the extent to which measures match with researchers’ theoretical intent, and thoroughly discuss how unmeasured confounding and unexpected meaning in measures might twist or reinforce the interpretation of findings.

## Limitations

There are some limitations to our approach. The first limitation is that most reviews only looked at English language publications (appendix 10). However, reviews that included studies written in other languages (e.g., Benton et al. which included English, Spanish and Portuguese language studies only found 2 of 43 studies were non-English; Chambers et al. imposing no language restrictions identified no studies published outside of English language journals) indicated that the language restriction is unlikely to be a major source of bias. Secondly, regarding the heterogeneity of designs and data collection, neither of included reviews nor this umbrella review conducted a formal meta-analysis. Thirdly, we excluded studies conducting SNA of personal relationship or provider friendship using social media, which theoretically contained some professional advice-seeking information and might be missing from this paper. Finally, no insight into mathematical theorems was provided as this was beyond the scope of this umbrella review. More arithmetical or geometrical information is available in related methodological texts like Borgatti et al. [48].

## Conclusions

There was a marked diversity across included articles in terms of reviewing purposes, participants and range of screening time, which indicated the necessities of this umbrella review for readers considering employing SNA and utilizing SNA results. This umbrella review presents, to our knowledge, the most comprehensive overview of provider networks and acts as a centralized repository of information for researchers and policymakers who are considering to enhance care delivery with an SNA-based approach. This review answered the questions: what the current state of knowledge regarding SNA of provider networks was, what constrained the utilization of SNA in healthcare settings, and what future research should seek to go beyond. The potential for broader utilization of SNA among providers in practice remains largely untapped. The findings of this review may contain important value for building optimal healthcare delivery networks and proposing pathways to the next step for the research community.

## Supplementary information

Additional file 1 Appendix 1-9.

Additional file 2 Appendix 10.

Additional file 3 Appendix 11 citation matrix.

Additional file 4 Appendix 12 PRISMA-Checklist.

Additional file 5 Appendix 14.

## Data Availability

All data generated or analyzed during this study are included in this published article and its electronic supplementary material.

## Abbreviations

SNA: SNA social network analysis
PRISMA: Preferred Reporting Items for Systematic Reviews and Meta-Analyses
AMSTAR: A Measurement Tool used to Assess systematic Reviews
CCA: Corrected Covered Area
LMIC: Low- and middle-income countries
SABM: stochastic actor-based network models
ERGM: exponential random graph models
QAP: quadratic assignment procedure
