# Supplementary material for: Review of social networks of professionals in healthcare settings—where are we and what else is needed?
Source: Global Health. 2021 Dec 4;17:139. doi: 10.1186/s12992-021-00772-7 (PMC8642762; doi:10.1186/s12992-021-00772-7)
Supplement: Supplementary file 1 — Appendix 1-9. [file 12992_2021_772_MOESM1_ESM.docx]

## Appendix 1: Search strategies

Notes：

We defined “health care professionals” as physicians, physician’s assistants, pharmacists, clinical officers, nurses, and others who provide health-related services to patients in formal medical settings. The search concept for SNA was adapted from the Chambers et al.’s and Sabot et al.’s systematic review of SNA^[[1]](#footnote-1),^^[[2]](#footnote-2)^. We used pearl growing techniques to identify the list of keywords and to develop the search strategies^[[3]](#footnote-3)^.

| No. | Search date | Database | Search strategies | No. found |
| --- | --- | --- | --- | --- |
| 1 | 8/31/2020 † | Pubmed | (((Interprofessional Relations[MeSH Terms]) AND (network*[Title/Abstract])) OR ("social network*"[Title/Abstract]) OR ("network theor*"[Title/Abstract]) OR ("network stud*"[Title/Abstract]) OR ("network intervention*"[Title/Abstract]) OR (“network structure*”[Title/Abstract]) OR ((("Sociometric Techniques"[MeSH Terms]) OR (Sociometr*[Title/Abstract]) OR (Sociogram*[Title/Abstract]) OR ("social map*"[Title/Abstract]) OR ("opinion leader*"[Title/Abstract])) AND (network*[Title/Abstract]))) AND ((Allied Health Personnel[MeSH Terms]) OR (Medical Staff Hospital[MeSH Terms]) OR (Clinician*[Title/Abstract]) OR (physician*[Title/Abstract]) OR (doctor*[Title/Abstract]) OR (pharmacist*[Title/Abstract]) OR (nurse*[Title/Abstract]) OR ("health care professional*"[Title/Abstract]) OR ("healthcare professional*"[Title/Abstract]) OR ("health professional*"[Title/Abstract]) OR ("healthcare provider*"[Title/Abstract]) OR ("health care provider*"[Title/Abstract]) OR (therapist* [Title/Abstract]) OR ("allied health"[Title/Abstract]) OR (“health care team*”[Title/Abstract]) OR (“healthcare team*”[Title/Abstract]) OR (“healthcare setting*”[Title/Abstract]) OR (“health care setting*”[Title/Abstract]) OR (“health care organi*”[Title/Abstract]) OR (“healthcare organi*”[Title/Abstract]) OR (“health sector*”[Title/Abstract])) AND (review*[Title/Abstract]) | 442 |
| 2 | 8/31/2020 † | EMBASE | #1 'social network*':ab,ti OR 'network theor*':ab,ti OR 'network stud*':ab,ti OR 'network intervention*':ab,ti OR 'network structure*':ab,ti | 30615 |
|  |  |  | #2 sociometr*:ab,ti OR sociogram*:ab,ti OR 'social map*':ab,ti OR 'opinion leader*':ab,ti | 3022 |
|  |  |  | #3 network*:ab,ti | 595745 |
|  |  |  | #4 #2 AND #3 | 354 |
|  |  |  | #5 #1 OR #4 | 30778 |
|  |  |  | #6 clinician*:ab,ti OR 'health care professional*':ab,ti OR 'healthcare professional*':ab,ti OR 'health professional*':ab,ti OR 'healthcare provider*':ab,ti OR 'health care provider*':ab,ti OR therapist*:ab,ti OR physician*:ab,ti OR doctor*:ab,ti OR pharmacist*:ab,ti OR nurse*:ab,ti OR 'allied health':ab,ti OR 'health care team*':ab,ti OR 'health care organi*':ab,ti OR 'healthcare organi*':ab,ti OR 'healthcare setting*':ab,ti OR 'health sector*':ab,ti OR 'healthcare team*':ab,ti OR 'health care setting*':ab,ti | 1538588 |
|  |  |  | #7 'health care personnel'/exp | 1591399 |
|  |  |  | #8 #6 OR #7 | 2491860 |
|  |  |  | #9 review*:ab,ti | 2761201 |
|  |  |  | #10 #5 AND #8 AND #9 | 639 |
| 3 | 8/31/2020 † | SCOPUS | ( TITLE-ABS-KEY( "social network*" ) OR TITLE-ABS-KEY ( "network theor*" ) OR TITLE-ABS-KEY ( "network stud*" ) OR TITLE-ABS-KEY ( "network intervention*" ) OR TITLE-ABS-KEY ( "network structure*" ) OR ( ( TITLE-ABS-KEY ( sociometr*) OR TITLE-ABS-KEY ( sociogram* ) OR TITLE-ABS-KEY ( "social map*" ) OR TITLE-ABS-KEY ( "opinion leader*" ) ) AND TITLE-ABS-KEY ( network* ) ) ) AND ( TITLE-ABS-KEY ( clinician* ) OR TITLE-ABS-KEY ( physician* ) OR TITLE-ABS-KEY ( doctor* ) OR TITLE-ABS-KEY(pharmacist*) OR TITLE-ABS-KEY ( nurse* ) OR TITLE-ABS-KEY ( "health care professional*" ) OR TITLE-ABS-KEY ( "healthcare professional*" ) OR TITLE-ABS-KEY ( "health professional*" ) OR TITLE-ABS-KEY ( "healthcare provider*" ) OR TITLE-ABS-KEY ( "health care provider*" ) OR TITLE-ABS-KEY ( therapist* ) OR TITLE-ABS-KEY ( "allied health" ) OR TITLE-ABS-KEY ( "health care team*" ) OR TITLE-ABS-KEY ( "healthcare team*" ) OR TITLE-ABS-KEY ( "healthcare setting*" ) OR TITLE-ABS-KEY ( "health care setting*" ) OR TITLE-ABS-KEY ( "health care organi*" ) OR TITLE-ABS-KEY ( "healthcare organi*" ) OR TITLE-ABS-KEY ( "health sector*" ) ) AND TITLE-ABS-KEY( review*) | 1127 |
| 4 | 8/31/2020 † | ProQuest | ((mainsubject.Exact(“Interprofessional Relations”) AND ab(network*)) OR ab("social network*") OR ab("network theor*") OR ab("network stud*") OR ab("network intervention*") OR ab(“network structure*”) OR ((mainsubject.Exact("Sociometric Techniques") OR ab(Sociometr*) OR ab(Sociogram*) OR ab("social map*") OR ab("opinion leader*")) AND ab(network*))) AND (mainsubject.Exact(“Allied Health Personnel”) OR ab(Clinician*) OR ab(physician*) OR ab(doctor*) OR ab(pharmacist*) OR ab(nurse*) OR ab("health care professional*") OR ab("healthcare professional*") OR ab("health professional*") OR ab("healthcare provider*") OR ab("health care provider*") OR ab(therapist*) OR ab("allied health") OR ab(“health care team*”) OR ab(“healthcare team*”) OR ab(“healthcare setting*”) OR ab(“health care setting*”) OR ab(“health care organi*”) OR ab(“healthcare organi*”) OR ab(“health sector*”)) AND ab(review*) | 665 |
| 5 | 8/31/2020 † | Web of Science Core Collection | (AB=( "social network*" OR "network theor*" OR "network stud*" OR "network intervention*" OR "network structure*" ) OR (AB=( sociometr* OR sociogram* OR "social map*" OR "opinion leader*" ) AND AB=( network* ) ) ) AND AB=( clinician* OR physician* OR doctor* OR pharmacist* OR nurse* OR "health care professional*" OR "healthcare professional*" OR "health professional*" OR "healthcare provider*" OR "health care provider*" OR therapist* OR "allied health" OR "health care team*" OR "healthcare team*" OR "healthcare setting*" OR "health care setting*" OR "health care organi*" OR "healthcare organi*" OR "health sector*" ) AND AB=( review*)  Choose English | 276 |
| 6 | 8/31/2020 † | Science Direct | ("social network!" OR "network theor!" OR "network stud!" OR "network intervention!") AND (clinician! OR physician! OR doctor! OR nurse! OR pharmacist!)  Choose review article | 404 |
| 7 | 8/31/2020 † | SAGE | [Abstract review]  And  [Abstract clinician OR therapist OR physician OR doctor OR pharmacist OR nurse OR health]  And  [Abstract "social network" OR "network theory" OR "network study" OR "network intervention" OR "network structure"] | 3 |
| 8 | 8/31/2020 † | WILEY | "clinician OR physician OR doctor OR pharmacist OR nurse OR health" in Abstract and ""social network" OR "network theory" OR "network study" OR "network intervention" OR "network structure"" in Abstract and "review" in Abstract | 121 |
| 9 | 8/31/2020 † | Grey literature: Google Scholar | allintitle: (clinician OR therapist OR physician OR doctor OR pharmacist OR nurse OR health）AND (network) AND (review)  exclude：patent, citation | 68 |

†: Updated 4/30/2021, no additional articles identified

## Appendix 2 Modified AMSTAR checklist

The AMSTAR has adequate face and content validity to measure quality of systematic reviews. However, the AMSTAR faces challenges when assessing the quality of methodology reviews. Consequently, this study used 11 key questions of AMSTAR as the basis to develop a generic tool applicable for assessing the quality of SNA reviews by slightly modifying or reinterpreting prompts and/or notes while preserving the original intent of checklist items. We did not exclude any study or finding weighted on the basis of quality evaluation, ascribing to the theory that some ponderable qualitative insights within individual studies may only emerge during the synthesis instead of the appraisal process.^[[4]](#footnote-4)^

**Notes**

• 1 POINTS: Statistical points should be allocated for each positive answer

(“Yes”)

• 0 POINTS: Towards other alternative answers (negative characteristics).

(“No”, “Can’t answer”, “Not applicable”)

• Maximum score of 11 POINTS for a perfect quality review.

**Modified AMSTAR checklist**

**1. Was an 'a priori' design provided?**

The research question and inclusion criteria should be established before the conduct of the review.

*Note: Need to refer to a protocol, methods, search strategy, or pre-determined/a priori published research objectives to score a “yes.”*

□ Yes

□ No

□ Can't answer

□ Not applicable

**2. Was there duplicate study selection and data extraction?**

There should be at least two independent data extractors and a consensus procedure for disagreements should be in place.

*Note: 2 people do study selection, 2 people do data extraction, consensus process or one person checks the other’s work.*

□ Yes

□ No

□ Can't answer

□ Not applicable

**3. Was a comprehensive literature search performed?**

At least two electronic sources should be searched. The report must include years and databases used (e.g., EMBASE and MEDLINE). Key words and/or MESH terms must be stated and where feasible the search strategy should be provided.

*Note: If at least 2 sources + keyword and/or strategy used, select “yes” (a grey literature search counts as supplementary).*

□ Yes

□ No

□ Can't answer

□ Not applicable

**4. Was the status of publication (i.e. grey literature) used as an inclusion criterion?**

□ Yes

□ No

□ Can't answer

□ Not applicable

The authors should state that they searched for reports regardless of their publication type. The authors should state whether or not they excluded any reports (from the systematic review), based on their publication status, language etc.

*Note: If review indicates that there was a search for “grey literature” or “unpublished literature,” indicate “yes.” Single database, dissertations, conference proceedings are all considered grey for this purpose. If searching a source that contains both grey and non-grey, must specify that they were searching for grey/unpublished lit.*

□ Yes

□ No

□ Can't answer

□ Not applicable

**5. Was a list of studies (included and excluded) provided?**

A list of included and excluded studies should be provided.

*Note: Acceptable if the excluded studies are referenced and/or total number is presented in a descriptive or diagram format e.g., PRISMA diagram.*

**6. Were the characteristics of the included studies provided?**

In an aggregated form such as a table, a summary of the reviewed results obtained from the original studies should be provided (e.g. measures used for SNA) alongside references pointing out studies assessed (e.g. numerical reference).

*Note: Acceptable if not in table format as long as they are described as above.*

□ Yes

□ No

□ Can't answer

□ Not applicable

**7. Was the scientific quality of the included studies assessed and documented?**

'A priori' methods of assessment should be provided using scoring tool or checklists to evaluate the quality of assessed studies; for other types of studies alternative items will be relevant.

*Note: Can include use of a quality scoring tool or checklist or a description of quality items, with some kind of result for EACH study (“low” or “high” is fine, as long as it is clear which studies scored “low” and which scored “high”; a summary score/range for all studies is not acceptable).*

□ Yes

□ No

□ Can't answer

□ Not applicable

**8. Was the scientific quality of the included studies used appropriately in formulating conclusions?**

The results of the methodological rigor and quality should be considered in the analysis/discussion and the conclusions of the review, and explicitly stated in formulating recommendations.

*Note: Might say something such as “the results should be interpreted with caution due to poor quality of included studies”; Cannot score “yes” for this question if scored “no”*

*for question 7.*

□ Yes

□ No

□ Can't answer

□ Not applicable

**9. Were the methods used to combine the findings of studies appropriate?**

For the pooled results, a comparison assessment should be done to ensure the studies were combinable, to assess its diversity (i.e., evaluate different type of social network being used in a table or descriptive format to allocate diversity in the results presented). The appropriateness of method used to combine results should be taken into consideration (i.e., is it sensible to combine?).

*Note: Indicate “yes” if a method is used to allocate diversity in the results presented*

*and suitable for the research question assessed (e.g. the type of* social network *applied in healthcare).*

□ Yes

□ No

□ Can't answer

□ Not applicable

**10. Was the likelihood of publication bias assessed?**

An assessment of publication bias can be accepted if it’s being accessed via using quality assessment test (e.g. scoring tool, checklists) and / or presented in the discussion by authors highlighting state (being bias or not) of articles assessed.

*Note: If no quality assessment test being included and / or articles’ bias state is not discussed, score “no”. Score “yes” if mentions that publication bias could not be assessed because there were fewer than 10 included studies.*

□ Yes

□ No

□ Can't answer

□ Not applicable

**11. Was the conflict of interest included?**

Potential sources of support should be clearly acknowledged in the systematic review.

*Note: To get a “yes,” must indicate source of funding or support for the systematic review.*

□ Yes

□ No

□ Can't answer

□ Not applicable

## Appendix 3: Citation matrix and calculation formulae

Illustrative example

|  | Review1 | Review2 | Review3 | Review4 |
| --- | --- | --- | --- | --- |
| Primary publication | 🗸 |  |  | 🗸 |
| Primary publication | 🗸 | 🗸 | 🗸 |  |
| Primary publication | 🗸 |  | 🗸 |  |
| Primary publication |  |  |  | 🗸 |

$$CA (covered area)=\frac{N}{\mathrm{rc}}$$

$$CCA (corrected covered area) =\frac{N-r}{rc-r}$$

CCA(%)=0–5, slight; 6–10, moderate; 11–15, high; > 15, very high overlap

Where N is the number of included publications (including double counting, this is the sum of ticked boxes in the citation matrix); where r is the number of rows (number of index publications) and c is the number of columns (number of reviews). For this illustrative example, CA= (3+1+2+2) / (4*4) = 50.0%; CCA= (8-4) / (16-4) = 33.3%

The citation matrix of primary publications of this umbrella review was presented in **Appendix 11**.

The overlap of this umbrella review:

CA=N/(rc) = (27+49+139+6+25+4+28+43+85+26+52+3+13) / (330*13) = 500/4290 = 11.7%

CCA = (N-r) / (rc-r) = (500-330) / (4290-330) = 170/3960 = 4.3%

## Appendix 4 Quality assessment results using the modified AMSTAR checklist.

| **No.** | **Article** | **Q1**  Was an ‘a priori design provided?’ | **Q2**  Was there duplicate study selection and data extraction? | **Q3**  Was a comprehensive literature search performed? | **Q4**  Was the status of publication (grey literature) used and inclusion criterion? | **Q5**  Was a list of studies (included and excluded) provided? | **Q6**  Were the characteristic of the included studies provided? | **Q7**  Was the scientific quality of the included studies assessed and documented? | **Q8**  Was the scientific quality of the included studies used appropriately to formulate conclusion? | **Q9**  Were the methods used to combine the findings of studies appropriate? | **Q10**  Was the likelihood of publication bias assessed?  (Any techniques used to prevent bias) | **Q11**  Was the conflict of interest included? |
| --- | --- | --- | --- | --- | --- | --- | --- | --- | --- | --- | --- | --- |
| 1 | Glegg *et al*. (2019) | YES  ==========  It is presented clearly at the question, search strategy and inclusion criteria. | YES  ==========  More than 1 reviewer | Yes | NO  ==========  Not stated. | Yes | Yes | NO  ==========  Quality assessment of articles is not being evaluated. | CAN’T ANSWER  ==========  Quality assessment is not conducted. | Yes | Yes  ==========  Mentioned bias and results should be interpreted with caution | Yes  ==========  Source of funding is displayed |
| 2 | DuGoff *et al*. (2018) | YES  ==========  It is presented clearly at the question, search strategy and inclusion criteria. | YES  ==========  More than 1 reviewer. | Yes | NO  ==========  Not stated. | Yes | Yes | NO  ==========  Quality assessment of articles is not being evaluated. | CAN’T ANSWER  ==========  Quality assessment is not conducted. | Yes | Yes  ==========  Mentioned bias and results should be interpreted with caution | Yes  ==========  Source of funding is displayed |
| 3 | Brunson *et al*. (2018) | YES  ==========  It is presented clearly at the question, search strategy and inclusion criteria. | NO  ==========  Not stated. | Yes | YES  ==========  52 conference presentations (papers and extended abstracts), 9 book sections, and 1 electronic preprint | Yes | Yes | NO  ==========  Quality assessment of articles is not being evaluated. | CAN’T ANSWER  ==========  Quality assessment is not conducted. | Yes | NO  ==========  Not stated. | Yes  ==========  No source of funding was gained |
| 4 | Sabot *et al*. (2017) | YES  ==========  It is presented clearly at the question, search strategy and inclusion criteria. | YES  ==========  More than 1 reviewer | Yes | Yes  ==========  Grey literature was searched via Popline. | Yes | Yes | Yes | Yes | Yes | Yes  ==========  Mentioned in limitations | Yes  ==========  Source of funding is displayed |
| 5 | Poghosyan *et al*. (2016) | YES  ==========  It is presented clearly at the question, search strategy and inclusion criteria. | YES  ==========  More than 1 reviewer | Yes | Yes  ==========  Google Scholar was used to identify additional studies | Yes | Yes | YES  ==========  The studies were assessed for quality before the data extraction | NO  ==========  Not stated. | Yes | Yes  ==========  Mentioned in limitations | NO  ==========  Source of funding is not displayed  **No conflict of interest statement** |
| 6 | Mitchell, *et al*. (2016) | YES  ==========  It is presented clearly at the question, search strategy and inclusion criteria. | YES  ==========  More than 1 reviewer | Yes | NO  ==========  Not stated. | Yes | Yes | NO  ==========  Quality assessment of articles is not being evaluated. | CAN’T ANSWER  ==========  Quality assessment is not conducted. | Yes | Yes  ==========  Mentioned in discussion | Yes  ==========  No source of funding was gained |
| 7 | Bae *et al*. (2015) | YES  ==========  It is presented clearly at the question, search strategy and inclusion criteria. | YES  ==========  More than 1 reviewer | Yes | NO  ==========  Not stated. | Yes | Yes | Yes | Yes  =======  Stated in discussion | Yes | NO  ==========  Not stated. | NO  ==========  Source of funding is not displayed  **No conflict of interest statement** |
| 8 | Benton *et al*. (2015) | YES  ==========  It is presented clearly at the question, search strategy and inclusion criteria. | YES  ==========  More than 1 reviewer | Yes | Yes  ==========  Google and Yahoo search engines were used to identify additional grey literature | Yes | Yes | NO  ==========  Quality assessment of articles is not being evaluated. | CAN’T ANSWER  ==========  Quality assessment is not conducted. | Yes | Yes  ==========  Mentioned in limitations | Yes  ==========  No source of funding was gained |
| 9 | Tasselli *et al*. (2014) | YES  ==========  It is presented clearly at the question, search strategy and inclusion criteria. | NO  ==========  Not stated. | Yes | Yes  ==========  Google Scholar was used | Yes | Yes | NO  ==========  Quality assessment of articles is not being evaluated. | CAN’T ANSWER  ==========  Quality assessment is not conducted. | Yes | NO  ==========  Not stated. | Yes  ==========  No source of funding was gained |
| 10 | Cunningham *et al*. (2012) | YES  ==========  It is presented clearly at the question, search strategy and inclusion criteria. | YES  ==========  More than 1 reviewer | Yes | No  ==========  not include the ‘grey literature’ as it did not meet the quality criteria of being peer reviewed and published in scholarly journals. | Yes | Yes | YES  ==========  By drawing on published checklists, quality was assessed | Yes  =======  Stated in results | Yes | NO  ==========  Not stated. | Yes  ==========  Source of funding is displayed |
| 11 | Chambers *et al*. (2012) | YES  ==========  It is presented clearly at the question, search strategy and inclusion criteria. | YES  ==========  More than 1 reviewer | Yes | Yes  ==========  Also searched the website of the International Network for Social Network analysis and Conference Proceedings Citation Index- Social Science & Humanities | Yes | Yes | YES  ==========  Quality (risk of bias) of level I studies was assessed by two authors (DC and PW) independently using the criteria of the Cochrane EPOC | Yes  =======  Stated in results | Yes | Yes  ==========  Mentioned in discussion | Yes  ==========  Source of funding is displayed |
| 12 | Dunn *et al*. (2011) | YES  ==========  It is presented clearly at the question, search strategy and inclusion criteria. | NO  ==========  Not stated. | No | No | No | Yes | NO  ==========  Quality assessment of articles is not being evaluated. | CAN’T ANSWER  ==========  Quality assessment is not conducted. | Yes | NO  ==========  Not stated. | Yes  ==========  Source of funding is displayed |
| 13 | Braithwaite *et al*. (2010) | YES  ==========  It is presented clearly at the question, search strategy and inclusion criteria. | YES  ==========  More than 1 reviewer | Yes | NO  ==========  Not stated. | Yes | Yes | NO  ==========  Quality assessment of articles is not being evaluated. | CAN’T ANSWER  ==========  Quality assessment is not conducted. | Yes | NO  ==========  Not stated. | Yes  ==========  Source of funding is displayed |

##
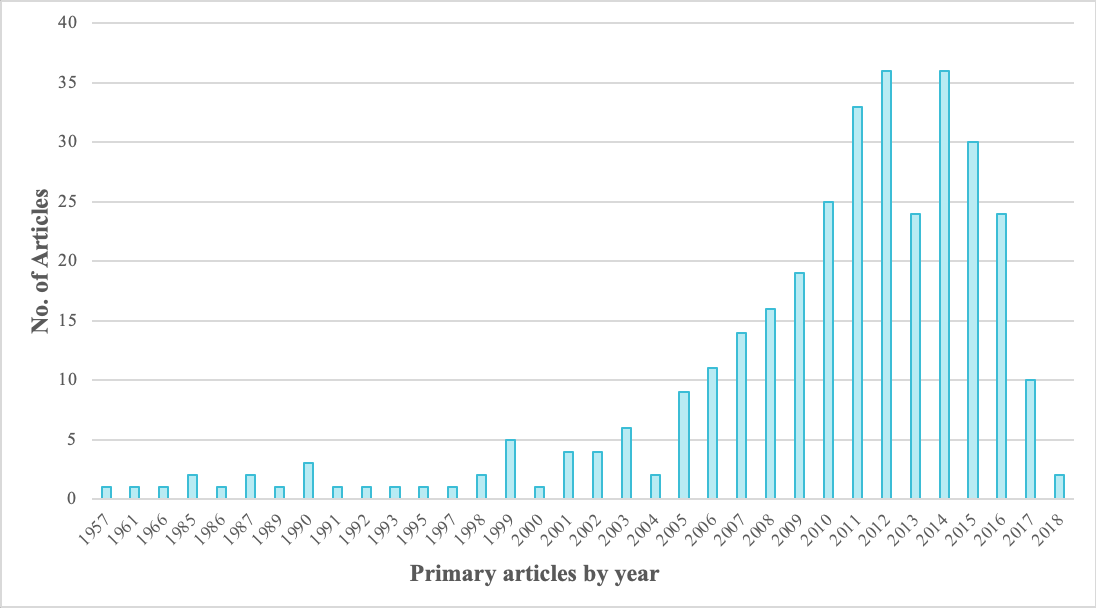
Appendix 5 Number of primary articles each year in included reviews, using the earliest date known to have been available.

##
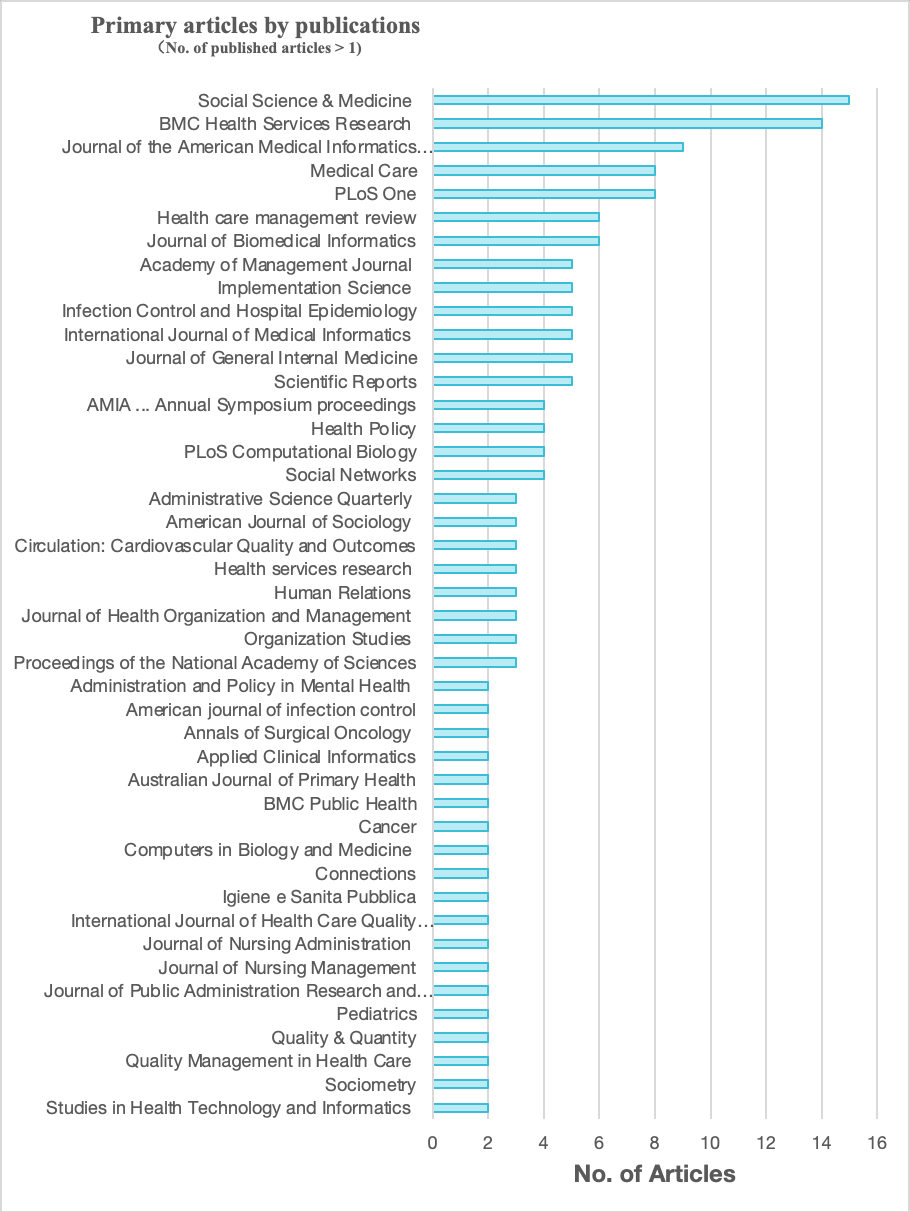
Appendix 6 Number of primary articles by publications in included reviews.

## Appendix 7 Relationships between Antecedents and Network Features

| **Review** | **factors** | **Findings** | |
| --- | --- | --- | --- |
| 1. Glegg et al. (2019) | | | |
|  | Individual and professional characteristics: gender, professional role or administrative role or Rank (i.e., leadership role), clinical experience, medical school, affiliation to formal groups, external communication (# visits from drug representatives), research orientation (# publications) | Attributes, such as research versus clinical productivity, professional field or specialty, leadership role and organizational prestige, as well as the presence of other types of ties (e.g., friendship, expertise recognition, previous collaborations), appear to be predictive or explanatory factors for the formation of information seeking or research collaboration ties. Conflicting findings regarding the influence of evidence informed practice (EIP) attitudes, experience, gender, and geographical proximity on tie formation were identified. For more information about the full array of correlational, dependent and independent variables and covariates (both relational variables and attributes) identified in the included studies, refer to Glegg et al. (2019) | |
|  | environmental characteristics: size of clinical practice, organizational division, occupational distance among members, geographical region |  |  |
|  | social attributes：perceived reputation |  |  |
|  | patient characteristics：age, sex, race, health status, intensity of care |  |  |
| 2. DuGoff et al. (2018) | | | |
|  | Researchers employed a range of different statistical approaches that varied widely in the number and types of included covariates that may confound the associations, which may explain why studies examining the same measure sometimes disagreed. | | |
| 5. Poghosyan et al. (2016) | | | |
|  | demographic characteristics | Conflicting reports regarding ties between members of similar age  Connections between physicians of similar gender | |
|  | professional affiliations | Members interact with others of the same discipline/subspecialty Information, advice, or guidance networks around individuals from certain professions | |
|  | characteristics of the patient populations served | Connections between physicians sharing patients Connections between physicians with similar patient populations | |
|  | structural factors such as geographic proximity (e.g. colleagues located in the same practice) | Connections between members working in physical or geographic proximity or in certain settings Connections between physicians with similar schedule or allocated time | |
| 7. Bae et al. (2015), most common factors: (1) the health professional characteristics; (2) setting/patient type. | | | |
|  | Length of care | Team coreness, how often the team worked together | Positive |
|  | Start time (later in the day) | Team coreness | Negative |
|  | Registered nurses | Team coreness | Positive |
|  | Operating room structure (centralized) | Operating room interdependency | Positive |
|  | Older age | Centrality and clique of health care professional relationship network | Positive |
|  | Physicians, Working transversal units | Centrality, prestige, and clique of health care professional relationship network | Positive |
|  | Within professional groups | Medication-related advice seeking network | Positive |
|  | Within professional groups | Problem-solving network, medication advice network, socializing network | Positive |
|  | Between professional groups | Medication advice-seeking interactions | Negative |
|  | Within physicians in the same clinic, physicians having more weekly clinical sessions | Discussion network | Positive |
|  | Male, physicians, at the same hospital, in close geographic proximity, similar practice intensity, taking care or similar racial compositions, patient age and percentage of Medicaid patients, similar patients | Patient sharing network | Positive |
|  | Attitude toward evidence-based medicine, field of specialization, same affiliation, directorate, co-authorship | Professional network of collaborate–density | Positive |
|  | Years since graduation, managerial role, number of publications | Professional network of collaborate–density | Negative |
|  | Higher-prestige hospital, percent of time doing clinical work in higher prestige hospitals | Esteem–in-degree centrality | Negative |
|  | Number of journals read/month, prestige of medical school in higher-prestige hospitals | Esteem–in-degree centrality | Positive |
|  | critical nodes failure at one, two, or three level | Degree centrality, betweenness Centrality, nodal cognitive demand | Negative |
|  | Number of residents, number of nursing staff | Density of communication network, density of advice network | Negative |
|  | Number of PD patients (≥10) | Size (one-step reach), number of connection (ties), Two-step reach, indegree centrality, outdegree centrality, inreach centrality (2 steps), betweenness centrality | Positive |
|  | Number of PD patients (≥10) | Reach efficiency | Negative |
|  | Setting of care delivery (hospital setting) | Size (one-step reach), number of connection (ties), density, two-step reach, in-degree centrality, out-degree centrality, in-closeness centrality, out-closeness centrality, in-reach centrality (two steps), out-reach centrality (one step), betweenness centrality | Positive |
|  | Setting of care delivery (hospital setting) | Reach efficiency | Negative |
|  | Occupation group (nursing) | Density | Negative |
|  | Occupation group (nursing) | Degree centralization | Positive |
|  | Attending local chapter meeting regularly | In-degree centrality, eigenvector centrality | Positive |
|  | Worked longer in current position (≥5 years) | In-degree centrality, out-degree centrality, eigenvector centrality | Positive |
|  | Using national healthcare safety network | In-degree centrality | Positive |
|  | Affiliated with university | Out-degree centrality | Negative |
| 9. Tasselli *et al*. (2014) | | | |
|  | Demographic attributes | Diversity in gender affects centralization in physicians’ teams and discussion of professional knowledge  Similar findings emerge while taking into account diversity in age, seniority and professional background  The GPs’ decision to take part in collaborative networks is influenced by gender and number of enrolled patients | |
|  | professional groups | Doctors and nurses display different structural configurations of their social networks  In doctors’ networks, social control is based on the peers’ assessment of the degree of peer-to-peer knowledge transfer  Professionals tend to forge advice, communication, and friendship ties with members of their same professional group  Different professional groups (GPs and pharmacists) have different perceptions of the structure of their networks  Barriers between professional groups tend to inhibit interprofessional interaction patterns | |
|  | organizational arrangements | Organizational arrangements, including rituals, teamwork, part-time and the management of caseload, facilitate social interactions between professionals  Long-term care units and flexible vs. traditional schedules affect social networks between nurses and physicians  Organizational arrangements, including the recruitment of junior doctors, the encouragements of the role of opinion leaders, multidisciplinary training and the support of peer interactions between nurses, facilitate patterns of social interactions between distinct professions  Geographical proximity can affect communication density in social networks | |

## Appendix 8 Relationships between Network Features and Health-related Consequences

| **Review** | **Findings** | |  |
| --- | --- | --- | --- |
| 1. Glegg et al. (2019) | | | |
|  | The study authors’ discussions about the influences of these network properties were clearly linked to prominent theoretical perspectives. Sample hypotheses relating to tie formation included predictors, such as homophily, existing ties (leading to reciprocity), and having a formal mechanism within the organization for interacting. Further hypothesis examples included that higher professional status would be associated with more knowledge exchange, tie homophily (i.e., sharing the same profession with a connection) would be associated with greater knowledge transfer ease, the presence of brokers (bridgers) would be associated with an increase in the receipt of useful information, particularly to managers, and that greater connectivity, frequency of contact, homophily, the presence of a highly connected clinical coordinator, and being an opinion leader would be associated with an increase the use of best practices. For more information about the full array of correlational, dependent and independent variables and covariates (both relational variables and attributes) identified in the included studies, refer to Glegg et al. (2019) | | |
| 2. DuGoff et al. (2018) | | | |
|  | ***(1) Measures of coordination***  health care costs, health care utilization, quality of care, and outcomes: studies examining indicators of coordination such as PCP centrality, Bipartite Clustering, and Care Density were statistically significantly associated with lower spending and lower health care utilization as measured by shorter length of stay. Coordination was also associated with better quality care including fewer inappropriate medications, fewer hospital readmissions, and less emergency department use. Two studies found coordination was associated with better patient outcomes measured using lower mortality rates. Studies also reported negative and non-significant results: Casalino and colleagues found that the percent of PCPs in a network was associated with more ambulatory care sensitive condition hospitalizations, and betweenness centrality was not significantly associated with ambulatory care sensitive condition hospitalizations. DuGoff and colleagues did not find that area-level tie persistence (i.e., multiyear patient-sharing relationships) between PCPs and other physicians were statistically associated with hospital readmissions rates. | | |
|  | ***(2) Measures of fragmentation***  degree for physicians in a network at the physician network level was significantly associated with higher spending and higher preventable hospitalization rates. In addition, using Medicaid claims data, Stein and colleagues found the likelihood that patients with an opioid use disorder would receive a prescription for an opioid or benzodiazepine increased with the number of physician communities seen. In a study of prostate cancer care, Pollack and colleagues found the relationship between urologist degree and prostatectomy complications to vary by city. | | |
|  | ***(3) Social contagion***  The study of peer-group effects on physician practice is an emerging area of study using social network tools. Pollack and colleagues found that a surgeon’s peer group use of brachytherapy—an adjuvant radiotherapy—and imaging studies influenced surgeons’ use in a later time period | | |
|  | ***(4) Network characteristics***  Studies examining network substructures suggest that there is a relationship between the organization of provider patient-sharing networks and patient outcomes. Uddin and colleagues also found that the presence of multiple physician communities within a hospital’s physician network was indicative of lower readmission rates | | |
|  | ***(5) Hospital acquired infections***  These studies found that patient transfers contribute to the spread of infectious diseases including MRSA, *Carbapenem-Resistent Enterobacteriaceae*, and *C. diff* | | |
| 4. Sabot et al. (2017) | | | |
|  | Patient outcomes generally improved when healthcare worker communication was denser and more centralized as measured by various centrality metrics. However, for both metric studies reported no significant association with some patient outcomes, as such more studies are needed to clarify patterns. | | |
| 5. Poghosyan et al. (2016) | | | |
|  | ***(1) Team member practice and outcomes***  Central network members influence others’ adoption of new treatments, prescriptions, or technology Subgroup membership is predictive of clinician behaviour | | |
|  | ***(2) Patient outcomes***  Different networks impact different patient outcomes Interconnected networks have better outcomes compared to unconnected networks | | |
|  | ***(3) Organizational outcomes***  Hierarchical network structures improve efficiency of care and hospital coding performance  Network members who regularly work together or consistently care for a particular patient have better outcomes Peripheral network members with more external connections are critical for improving collaboration with institutions | | |
| 6. Mitchell et al. (2016) | | | |
|  | The high density in 2 staff network studies was associated with the cooperation needed to provide care to residents with dementia. Staff's boundary-spanning led to higher-status nurses becoming more involved in decision-making and problem-solving in one study. In another, the outcome was staff treating residents with more respect and actively caring for them. | | |
| 7. Bae *et al*. (2015) | | | |
|  | Centrality of network location | Adoption and use of a computer-based hospital information system | Positive |
|  | Centrality of the referral and consultation network | Diffusion process | Positive |
|  | Central location in support network from coworkers and broad staff | Coping with stress | Positive |
|  | Median adjusted degree of network | Medicare spending, hospital days, physician visits | Positive |
|  | Betweenness centrality of network | Overall spending, spending on imaging and tests, physician visits, and Medical specialist visits | Negative |
|  | Communication relations and past experience | Learn to trust (distrust) | Positive |
|  | Communication network – clustering coefficient, cliques, triads | ADEs | Negative |
|  | Communication network–component count strong | Falls, symptom capacity | Negative |
|  | Communication network–component count weak, fragmentation, hierarchy, isolates | Symptom capacity | Negative |
|  | Communication network–density, diffusion, centrality in degree | Symptom capacity | Positive |
|  | Communication network–diffusion, eigenvector centrality | Simple self-care, complex self-care | Negative |
|  | Communication network–hierarchy | Simple self-care, complex self-care | Positive |
|  | Communication network–hierarchy | Well cared for–individual care | Negative |
|  | Communication network–eigenvector centrality | Well cared for–individual care | Positive |
|  | Social network–density | Coordination performance, coordination quality, flights | Negative |
|  | Social network–degree | Coordination performance, above average time, coordination quality, flights | Positive |
|  | Social network–network centralization | Flights | Negative |
| 9. Tasselli *et al*. (2014) | | | |
|  | **(1) s*atisfaction at work***  Network closure boosts social support and buffers in turn the negative effects of work stress on nurses’ self-rated health  But social support from network comembers is unrelated to job satisfaction for physicians and nurses in managerial roles  Professional experience and workload complement the role of social support in predicting physicians’ job satisfaction  Job satisfaction relates to the occupation of central positions in informal networks at work | | |
|  | ***(2) leadership***  Professionals who occupy central positions in social networks tend to be considered opinion leaders by their coworkers  Irrespective of network position, individual attributes (i.e., language and gender) and skills matter in predicting leadership emergence | | |
|  | ***(3) Professional behaviors***  Social interactions, network homogeneity, and the action of informal opinion leaders in social networks affect physicians’ prescribing behavior  Direct ties and structural importance in the advice network predict the physicians’ attitudes toward evidence-based medicine  Recruiting people who are perceived as opinion leaders by network comembers facilitates behavioral change | | |
|  | ***(3) knowledge transfer***  Weak ties with coworkers help professionals acquire useful knowledge  Brokerage allows actors to acquire and mobilize nonredundant knowledge and to initiate change initiatives  Network closure helps knowledge transfer between different professional groups and boosts multiprofessional interaction  Environmental constraints and organizational variables combine with network structure in predicting knowledge transfer patterns | | |
|  | ***(4) diffusion of innovation***  Network contagion has been found to predict the diffusion of a new drug in a community of physicians.  Data reanalyses showed that the adoption of the new drug among physicians could be explained by structural equivalence in social networks  Data reanalyses showed that advice and discussion networks did not have effects on adoption of the new drug.  Centrality in professionals’ social networks can explain timing of adoption of innovation  Peer interactions predict likelihood and timing of adoption  Barriers between distinct professions can retard the spread of innovation  Adoption of a new technology can predict in turn networking patterns, in terms of centrality and tie strength  Adoption of innovation in professional networks can also be contingent on organizational structure, ideological differences, and communication campaigns | | |
|  | ***(5) performance***  Network centrality tends to be positively associated with performance, although the relationship between centrality and performance is not univocal  But managers with the highest level of centrality tend to be unable to challenge the existing strategy with risk of lower performance  Network density is positively associated with performance at the team and organizational level  Centralization around a dominant actor is negatively related to performance  Ties linking physicians with the external environment are positively associated with performance  Social networks combine with work design in predicting outcomes  The structures of interpersonal connections between professionals explain higher level outcomes, including organizational performance | | |
| 10. Cunningham et al. (2012), health network quality and safety | | | |
|  | Brokerage | Important in bridging connections and obviating ‘structural holes’ in hospitals  Good coding performance is associated with a knowledge sharing network structure rich in brokerage and hierarchy, rather than density | |
|  | Centrality | Centrality of key organisations or actors in a network is important, and can be a strength or potential vulnerability for network sustainability  Directors of nursing are more central in their networks than clinical directors of medicine and their networks are more hierarchical—hence better adapted to gathering and disseminating information  The higher the centrality of the hospital in its network, the better the hospital performance | |
|  | Degrees of separation | Analysis of ‘degrees of separation’ can show the level of connectivity in a professional network | |
|  | Density | The denser the GP network the lower the variation in performance  Clinical directors of medicine are embedded in more densely connected networks (cliques), than directors of nursing, and can be stronger instruments for changing, or resisting changes, in clinical behaviour. Networks of directors of nursing have lower density, with advantages in accessing information | |
|  | Diffusion | Ideological tension can block the spread of knowledge and new work practices within the professional network  Gaps in the network of informal ties will impede the dissemination of information and the spread of social influence between nurse executives and physician leaders, while non-clinical managers have a brokerage role | |
|  | Homophily | People seek advice, or influence or discuss important professional matters with those similar to themselves (profession, gender, age, seniority), with implications for communication exchanges  Physician leaders have more extreme homophily than senior nurses | |
|  | Hierarchy | A large number of people in the network seek information from particular individuals  For health professional teams other than medicine, collaboration on problem-solving and decision-making is limited to higher status professionals  Nursing networks are more hierarchical than medical networks | |
|  | Integration and cliques | Relationships between groups of agencies, services or providers (cliques) in a network may be more important than the relationship between all agencies in the network  SNA can identify agencies and actors who are not well linked in the network | |
|  | Multiplexity | Employees forming a greater number of ties with co-workers are more embedded and have lower turnover | |
|  | Network roles | Individual roles in networks are important for communication and information dissemination: ‘broker’ and ‘bridging’ roles, ‘cliques’, ‘isolates’ | |
|  | Network stability | Network stability is related to network effectiveness, and can moderate the impact of resources  Longitudinal SNA can measure network expansion, with decreased fragmentation increasing potential information flow | |
|  | Reciprocity | Reciprocity of ties shows whether there is a hierarchical (low reciprocity) or horizontal (high reciprocity) structure in the professional network | |
|  | Social capital | Organisational social capital, in addition to professional experience and workload, can predict overall job satisfaction  Social influence of peer professionals has a greater impact than social capital on health professional performance | |
|  | Social climate | Positive social climate protects nurses against burnout  Professional and social networks and support do not mitigate against work stress of chief manager nurses or physician clinical directors | |

## Appendix 9 Inconsistent findings about Relationships among Antecedents, Network Features and Health-related Consequences

| **Antecedents or Consequences** | **Conflicting findings** | **Review** |
| --- | --- | --- |
| **Antecedents** | | |
| demographic characteristics of providers | - Conflicting findings regarding the influence of EIP attitudes, experience, gender, and geographical proximity on tie formation were identified. | 1. Glegg et al. (2019) |
|  | - Conflicting reports regarding ties between members of similar demographic characteristics. | 5. Poghosyan et al. (2016) |
| professional characteristics of providers | - A study conducted by Meltzer et al. (2010) observed clustering of attending physicians by specialty, such as geriatrics. These specialists created cohesive subgroups within the network and appeared less integrated into the set of internists, compared to their internist colleagues. However, Burt et al. (2012) found stronger ties between team members of different professions; specifically, physicians primarily developed personal and professional relationships with RNs. Moreover, physicians reported greater connections and trust with RNs when dealing with clinical and operational issues (Burt et al., 2012). | 5. Poghosyan et al. (2016) |
|  | - In a study of two medical intensive care units (ICUs), Lurie et al. (2009) found that physicians had more frequent communication in the team characterized by a large number of connections between team members, whereas the nursing staff was more central in the other team, characterized by a small number of connections between team members; certain team members dictated information flow through the network, on one team physicians and in another team nurses. In contrast, Benham-Hutchins and Effken (2010) reported that across four interdisciplinary teams, no professional group dominated communication. Rather, one or more influential providers from medicine, nursing, or pharmacy were central to team communication. |  |
|  |  |  |
| **Consequences** | | |
| (1) Influence on professional behaviors | - physician subgroup membership was associated with the likelihood of their patients undergoing a prostatectomy. Professional ties, however, have not had similar influence on clinician practice across all studies. For example, research conducted among attending physicians and residents demonstrated that physicians’ professional networks were not correlated with their electronic health records (HER) usage. | 5.Poghosyan et al. (2016) |
|  | - Impacts of professionals’ advice networks on adoption were found to be absent (Marsden & Podolny, 1990), then found to be significant (Strang & Tuma, 1993), and then again declared not significant (Van den Bulte & Lilien, 2001). | 9. Tasselli *et al*. (2014) |
| (2) organizational outcome and performance of coordination;(3) quality of care and patient outcomes; (4) health care utilization and costs. | Two studies found coordination was associated with better patient outcomes measured using lower mortality rates. Studies also reported negative and non-significant results: Casalino and colleagues found that the percent of PCPs in a network was associated with more ambulatory care sensitive condition hospitalizations, and betweenness centrality was not significantly associated with ambulatory care sensitive condition hospitalizations. DuGoff and colleagues did not find that area-level tie persistence (i.e., multiyear patient-sharing relationships) between PCPs and other physicians were statistically associated with hospital readmissions rates. | 2. DuGoff et al. (2018) |
|  | ***Fragmentation:***  degree for physicians in a network at the physician network level was significantly associated with higher spending and higher preventable hospitalization rates. In addition, using Medicaid claims data, Stein and colleagues found the likelihood that patients with an opioid use disorder would receive a prescription for an opioid or benzodiazepine increased with the number of physician communities seen. In a study of prostate cancer care, Pollack and colleagues found the relationship between urologist degree and prostatectomy complications to vary by city. | 2. DuGoff et al. (2018) |
|  | **C*oordinatio*n:**  Patient outcomes generally improved when healthcare worker communication was denser and more centralized as measured by various centrality metrics. However, for both metric studies reported no significant association with some patient outcomes, as such more studies are needed to clarify patterns. | 4. Sabot et al. (2017) |
|  | **C*oordinatio*n:**  Interconnected networks have better outcomes compared to unconnected networks  Peripheral network members with more external connections are critical for improving collaboration with institutions | 5. Poghosyan et al. (2016) |
|  | **C*oordinatio*n:**  The high density in 2 staff network studies was associated with the cooperation needed to provide care to residents with dementia. | 6. Mitchell et al. (2016) |
|  | ***Fragmentation:***  Social network features adversely related to outcomes are as follows. Betweenness centrality of network was negatively related to health care use (physician visits and Medical specialist visits; Barnett et al., 2012). Clustering and hierarchy of the communication network contributed to care outcomes adversely (e.g., symptom capacity, well cared for individual care, and adverse drug events; Effken et al., 2013). Density and centralization of the social network were negatively related to the performance and quality of coordination (Hossain & Guan, 2012).  The degree of social network was related to greater performance and quality of coordination (Hossain & Guan, 2012).  Density and centralization of the social network were negatively related to the performance and quality of coordination (Hossain & Guan, 2012) | 7. Bae *et al*. (2015) |
|  | Intuitively, a large network is supposed to provide professionals with performance pay-offs, because many contacts can represent an important vehicle conveying status and information (Peng et al., 2006). However, emerging research has shown that the connections between centrality and performance can be controversial (Chung et al., 2006). | 9. Tasselli *et al*. (2014) |

## Appendix 13: Utilization and potential of SNA regarding Healthcare Providers

| **Review** | **Utilization of SNA** |  | **Potential of SNA** |
| --- | --- | --- | --- |
| 1. Glegg et al. (2019) | - SNA is a valuable approach for evaluating key network characteristics, structures, and positions of relevance to knowledge translation (KT), implementation, and evidence informed practice. - Examining how network structure influences connections and the implications of those holding prominent network positions can provide insights to improve network-based KT processes. - A SNA perspective has been used to explore the patterns and efficiencies of information sharing within and across professions, to identify positions of influence and determine their effectiveness, to predict or to explain patterns of ties based on attributes or network structure, to compare the structural characteristics of different models of KT, and to examine the relationship between network properties and EIP attitudes and behaviors. Using a longitudinal approach, SNA has been used to evaluate the influence of social structure on knowledge exchange over time, as well as to evaluate network change following a quality improvement intervention. - Specifically, implementation happens within a complex system, and network approaches have been used to study complex systems; the link between the two areas demands greater attention. |  | - Social network analysis (SNA) has the potential to augment our understanding of knowledge translation (KT) (including implementation efforts) success by applying a network lens that examines the influence of relationships and social structures on research use and intervention acceptability by health professionals. - basic SNA can be used to identify key players with influence within the network; subsequent analyses can be used to explain how these individuals came to hold these positions. Knowledge of an individual’s structural position may also help to determine from whom they may seek evidence or KT support. This information can be used to develop KT interventions that target specific health professionals or groups of individuals based on their network structure or key attributes to strengthen KT processes. |
| 2. DuGoff et al. (2018) | - Social network tools can provide measures of health care provider relationships and the organizational context that affects health care delivery. - The use of social network tools to understand health care delivery is an emerging area of health services research spurred by recent delivery system reforms, including accountable care organizations and patient-centered medical homes. These models of care depend on fundamentally changing provider relationships to improve care coordination. |  | - Policymakers should consider using social network tools to monitor health care organizational context and changes. - A growing number of studies use patient-sharing relationships to construct physician networks, which offers opportunities to build on prior studies and harmonize methods. - Depending on their focus, authors suggested a wide range of possible applications from identifying high-performing networks of providers to redesigning hospital patient transfer processes. Studies of provider patient-sharing networks could also be used to inform other important policy issues with respect to the ideal composition of provider networks, the appropriate size of networks (e.g., when is a narrow network too narrow?), and how to most efficiently refer patients to specialty care. |
| 3. Brunson et al. (2018) | - Routinely collected health care data (RCHD) such as electronic medical records and public databases were identified as the most common sources of data and network analysis (NA) was identified as the most common modelling paradigm. - Fifty studies analyzed interorganizational health care systems as institutional exchange networks, the most common instantiation of which were patient-transfer and patient-sharing networks. - Another group of 33 studies modeled institutional and regional physician communities as physician collaboration networks. - A distinctive group of 59 studies concerned relational models of clinical events mined from patient- or encounter-level health records (clinical co-occurrence networks) most often used to generate etiological hypotheses. - The remaining 47 studies fit into a highly modular group investigating workplace interaction networks, including network models of shared record access, interprovider communication, interpersonal contacts, and patient handoffs. |  | - patient-sharing networks provided an exceptionally detailed setting in which the social diffusion of practice could be measured |
| 4. Sabot et al. (2017) | - Understanding and harnessing the power of existing professional advice networks among healthcare providers could assist in influencing provider practice and improving health outcomes in low- and middle-income countries (LMIC). |  | - Social network analysis (SNA) can lend insight into defining, measuring, and understanding these professional communication networks and therefore designing effective network interventions to improve provider performance and ultimately, health outcomes |
| 5. Poghosyan et al. (2016) | - Social network analysis (SNA), a sociometric method, is capable of identifying relational structures. It employs the use of graphical and statistical methods to characterize and analyze existing relationships between individuals as well as the overall network shape at the team level. Such evidence reveals what constitutes a productive team, how members collaborate, and how different relational structures affect quality of care and outcomes. In addition, it can also inform leveraging relational structures to promote information exchange, accelerate behavior change, and increase diffusion of evidence-based practices to improve individual and team performance as well as patient care and outcomes. |  | - Administrators and policy makers can use knowledge of health care networks to leverage relational structures in teams and tailor interventions that facilitate information exchange, promote collaboration, increase diffusion of evidence-based practices, and potentially improve individual and team performance as well as patient care and outcomes. - Managers in health care organizations should identify key actors as strategic members whom they can depend upon for quick diffusion of information and innovations. For example, as the health care organizations in the USA are implementing innovations such as Patient Centered Medical Homes or Accountable Care Organizations, that emphasize team- based care, these key actors can play a vital role (Bao et al., 2012). They can reach out to disconnected team members and share resources and information with them, which will subsequently improve teamwork and team-based care. - As policy makers are calling for developing optimal team-based models in the USA, administrators should identify isolated members in their teams and consider bridge-building activities between subgroups and individuals that do not share many ties, which could be due to professional differences. Also, the professional mix of teams can be modifiable by administrators. They can design teams with optimal professional mix to depending the clinical circumstances of patient populations to achieve best outcomes. - Administrators can consider factors such as how close team members are to each other, where central members are located, and how well all members are linked to each other. They also can leverage network structures to promote effective communication. |
| 6. Mitchell *et al*. (2016) | Health system reform in many countries has looked to network governance because of the advantages identified.   - For example, the English National Health Service funded a networks program of research on clinical networks starting in 2004. A review of 8 of these noted the “utility of network forms in tackling wicked problems.” - Introduction of a managed clinical network in Scotland, set up to improve care for people with diabetes, was effective in addressing collaboration and communication challenges across professional and organizational boundaries. The evaluation identified that the network had resulted in changes in professional practice that delivered a dramatic fall in the rates of hospital referrals and a major shift to primary care for people diagnosed with type 2 diabetes. The network also improved simpler processes faster and facilitated continuous improvement for complex processes. - Meltzer et al showed how SNA could be feasibly implemented to design effective quality improvement teams in the United States. |  | - In relation to clinical care, network structures could assist in the cost neutral roll-out of biopsychosocial approaches to care. |
| 7. Bae *et al*. (2015) | - In health care settings, social network analysis (SNA) has been used to understand communication and collaboration of health care providers, diffusion of new practice, and knowledge sharing among physicians. - SNA provides a means of mapping and exposing channels of communication and information flow between people in important groups within an organization. It explores the types of relationships that generate communication and learning, rather than focusing on the strength of individual relationships. - SNA allows the study of complex communication and interaction patterns in health care settings. Because communication and interaction among health care providers are crucial to improve patient safety and quality of care, SNA is an important analytic method that can help identify health care gaps affecting patient safety. |  | - Given increased emphasis of health care providers' teamwork and communication among team members, it is critical to understand the social network among health care providers and use them for patient safety and quality of care as well as better outcomes for health care providers' teamwork. |
| 8. Benton *et al*. (2015) | - Communication between team members is critical in the delivery of quality care. Social network analysis is increasingly being used to explore such communication. |  | - SNA does show considerable promise in offering insights into the way information flows between individuals, teams, institutions and other structures. An understanding of these structures would, at least in some cases, provide a means of improving team communication and associated quality of care . - Furthermore, the range of data sources observed – primary quantitative survey data, secondary analysis of survey and literature sources, as well as the use of semi-structured interviews – highlights the flexibility of applying this technique and may thereby facilitate triangulation of evidence from multiple sources when considering both practice and policy issues. |
| 9.Tasselli *et al*. (2014) | - At its best, social network analysis draws from traditions of research and theory in sociology and organization studies to describe how professionals build their social interactions at work and how professional networks affect outcomes of importance for both individuals and organizations. |  |  |
| 10. Cunningham *et al*. (2012) | - Effective professional networks employ natural structural network features (eg, bridges, brokers, density, centrality, degrees of separation, social capital, trust) in producing collaboratively oriented healthcare. This requires efficient transmission of information and social and professional interaction within and across networks. For those using networks to improve care, recurring success factors are understanding your network's characteristics, attending to its functioning and investing time in facilitating its improvement. - Social network analysis (SNA) can be used to examine structural relationships and influence in networks, the way information travels in networks, diffusion of innovative ideas, tools or practices, and sustainability of networks. It is the structure of networks and how the structural properties affect behaviour that is informative, not simply the characteristics of the network members. |  | - The evidence demonstrates that creating cohesive, collaborative networks (of professionals or agencies) can pay dividends in coordinating care and attending to quality and safety issues and agendas. - The presence of key players, often in management or leadership roles, who act as connectors to transmit information, bridge disparate groups, liaise across parts of networks and enable social and professional interaction is vital. |
| 11.Chambers *et al*. (2012) | - Social network analysis (SNA) offers a means of mapping and exposing the hidden channels of communication and information flow, collaboration and disconnects between people in strategically important groups within an organisation. Rather than focusing solely on the strength of individual relationships, it explores the types of relationships that condition communication and learning. - Research funded by the NIHR (National Institute for Health Research) Service Delivery and Organisation Programme and more recently the development of NIHR Collaborations for Leadership in Applied Health Research and Care (CLAHRCs), has refocused attention on the role of social interactions and networks in the ability of health service organisations to identify and exploit knowledge from outside the National Health Service (NHS). |  | - We are utilising SNA to inform the development and implementation of tailored behaviour-change interventions to improve the uptake of evidence into practice in the English National Health Service. |
| 12. Dunn et al. (2011) | - Social network analysis is an increasingly popular sociological method used to describe and understand the social aspects of communication patterns in the health care sector. |  |  |
| 13.Braithwaite *et al*. (2010) | - Gaps (i.e., the network holes, spaces and missing ties that create between-group problems and opportunities for their resolution) are typically regarded as a problem to be solved. People are stimulated to close or plug them. Researchers are moved to fill deficits in the literature in order to realise a more complete knowledge base, health authorities want to bridge policy-practice disconnections, managers to secure resources to remedy shortfalls between poor and idealised care, and clinicians to provide services to patients across the divides of organisational silos. - Despite practical and policy work in many health systems to bridge gaps, it is valuable to study research examining them for the insights provided. Structural holes, spaces between social clusters and weak or absent ties represent fissures in networks, located in less densely populated parts of otherwise closely connected social structures. Such gaps are useful as they illustrate how communication potentially breaks down or interactivity fails. |  | - Gaps (i.e., the network holes, spaces and missing ties that create between-group problems and opportunities for their resolution) offer insights into social structures, and how real world behaviours of participants in workplaces, organisations and institutions are fragile. The paper highlights the circumstances in which network disjunctures and group divides manifest. Knowledge of these phenomenon provides opportunities for working out ways to improve health sector organisational communications, knowledge transmission and relationships. |

## Appendix 14: Gaps and Suggestions about SNA regarding Healthcare Providers

| **Review** | **Gaps** |  | **Suggestions** |
| --- | --- | --- | --- |
| 1. Glegg et al. (2019) | - With more than half of the included studies examining physician-only networks, and only a handful studying interprofessional health care teams, great opportunity exists to expand the range of professions under study. Because of the growing shift in health services delivery from profession-based to collaborative practice models involving interprofessional teams [57], further research is needed to evaluate the generalizability of findings beyond physician networks, as well as in other health care contexts. - Because of its examination of information flow, predominantly, this body of research presents a narrow view of KT that focuses primarily at the individual level of evidence-based decision-making. This limitation relates in part to the scope of the review, as well as the consideration that other actors (e.g., health leaders, researchers) rather than health professionals may typically manage many of the KT activities that were not represented. |  | - The included articles focused on individual level evidence-based decision-making: we recommend also applying SNA to meso- or macro-level KT activities. SNA research that expands the range of professions under study, examines network dynamics over time, extends the depth of analysis of the role of network structure on KT processes and outcomes, and employs mixed methods to triangulate findings, is needed to advance the field. - Longitudinal research, a more representative range of populations, the use of interviews, document review and observation for data collection, greater depth of analysis, and the leveraging of network visualizations can augment the contributions of SNA to the KT science knowledge base. - Understanding how network properties can be used as proxies to measure social processes (e.g., information exchange, best practice adoption, decision-making, influence) can help KT scientists to apply SNA effectively to expand the range of measures that can be used to evaluate KT efforts. |
| 2. DuGoff et al. (2018) | - One gap centers on the actors used to construct the networks. Studies of providers focused on physicians and hospitals; only two studies included allied health professionals such as nurses and pharmacists. Given the importance of post-acute care in geographic variations in health care utilization, further work is needed to understand the interplay between post-acute care facilities and community-based physicians - Another area for further research is how patient-sharing networks change over time or in response to different incentives. Longitudinal studies may provide insight into the impact of policy reforms that seek to alter the ways in which patients receive care. - Finally, social network approaches are increasingly being deployed in public health initiatives [6, 68]. However, we did not identify studies that used patient-sharing networks to develop or evaluate an intervention. |  | - First, researchers should determine how many shared patients are necessary to form a tie between two nodes. - Second, researchers must determine which providers should be included or excluded. - Third, appropriate geographic boundaries need to be set for determining a provider network, which may influence the network’s structure. - Fourth, selecting an approach for identifying communities or clusters of providers may be challenging. We found Girvan-Newman’s algorithm to be the most popular, but other approaches are likely valid. |
| 3. Brunson et al. (2018) | - The network conceptual model is most illustratively called into question in the clinical co-occurrence setting: There are widely recognized problems with collapsing co-occurrence data to unipartite network models, but few studies of the “diseaseome” addressed them. - Several additional studies discussed biases in their data and tested the sensitivity of their results to different sources and thresholds, but these efforts were far narrower than the breadth of methods used, and none discussed concomitant differences in the resulting network structure. |  | - Some studies employed network measures without specific motivation, eg, degree and betweenness centrality as possible social determinants of methadone treatment continuation. In this case, a discernible effect of degree was given a reasonable interpretation, but an indiscernible effect of betweenness was not commented upon; had it been theoretically motivated, an account of this result would clearly be required. |
| 4. Sabot et al. (2017) | - Network methods are underutilized for the purposes of understanding professional communication and performance among healthcare providers. The paucity of articles meeting our search criteria, lack of studies in middle- and low-income contexts, limited number in non-tertiary settings, and few longitudinal, experimental designs, or network interventions present clear research gaps. - A general challenge generated by SNA methods is the need to clearly define the study boundary, which can limit sample size and therefore affect the broader generalizability. This came up in several studies noting areas for further research including broadening to other settings and repeating the study elsewhere given the limited sample size. The sample size limitation related less to the number of nodes, but more to the number and type of whole networks included. - The lack of longitudinal and experimental designs speaks to a broader challenge in the field as these are new areas for application of SNA methods, and the analytical tools and software are still in development. This limited the ability of SNA studies to address causal pathways. - Similarly, the limited qualitative methods being integrated into the studies constrain the contextual understanding of the network properties quantified and visualized through applying the quantitative SNA methods. |  | - One of the challenges facing researchers wanting to use SNA methods is the lack of validated SNA survey tools for use in the health sector, as highlighted by Creswick and Westbrook and Perkins et al. While this only is relevant for those interested in using sociometric survey methods, as more studies use SNA methods, we can anticipate that a set of tools or best practices for applying a range of SNA methods will emerge. |
| 5. Poghosyan et al. (2016) | - Team member demographic characteristics, professional affiliations, and clinical circumstances of patient populations can influence network formation. The findings were mixed regarding the influence of demographic characteristics. Some studies indicated that similarity in terms of age and gender may promote formation of the network while others did not find significant associations between these characteristics and network formation. - The review revealed many SNA studies conducted in other fields (e.g. sociology, management, engineering, etc.), the literature investigating health care team networks appears to be advancing slowly. |  | - None of the reviewed studies looked at the network dynamics over time. Network structures can be transient and will evolve over time as teams experience turnover, although such observations have yet to be tested longitudinally. Future research could focus on producing evidence about these changes to provide a deeper understanding about how networks regroup or fall apart and how their evolution affects patient care and outcomes. |
| 6. Mitchell *et al*. (2016) | - The review has highlighted that the adoption of an SNA to facilitate the improvement of care to residents in LTC has not yet made the same progress as in other health and clinical settings, where studies have linked network structure to evidence of outcomes. |  | - Further research into network structures could investigate how social networks improve staff's workplace experience, enable better care for residents, and contribute to organizational success in a constantly changing environment. |
| 7. Bae *et al*. (2015) | - Current studies on the social ties of health care workforce professionals include several assessments of inefficiencies. The level of technical sophistication in these studies tended to be low. - There is considerable risk for bias in the 28 included studies. There were no studies that used an intervention to attempt to change the structure or function of the social network and no designs that used experimental study design with a control group. |  | - Future study using enhanced sophistication in study design, analysis, and patient outcome testing are warranted to fully leverage the potential of SNA in health care studies. - To implement the interventions, researchers may need to change the current layout in health care settings and may have to use a longitudinal study design. SNA studies that involve altering the physical architecture of a unit present unique difficulty in finding appropriate study sites. |
| 8. Benton *et al*. (2015) | - So far it would appear that no author or group of authors have developed a programme of research in the nursing field using the social network analysis approach - The dominance of literature from North America may be viewed as problematic as the underlying structures and themes may be an artefact of cultural communication norms from this region. - The most significant limitation of the work conducted and published in the literature to this point relates to the one-off nature, single point-in-time and often small sample sizes exhibited by many of the studies. |  | - The lack of longitudinal studies and the absence of replication across multiple sites should be seen as an opportunity for further research. - In terms of how this technique might further contribute to our understanding of interventions on such factors as team communication or on the consequences of various learning interventions, more sophisticated designs would be required. However, the current dearth of multicentre and time-series designs could be viewed as a potential opportunity for further work. - There is a need to conduct more intervention-based studies where the underlying network structure is explored to gain insights into the outcomes of differing structures as well as the contribution that different actors play and how various intervention impact on network outcomes. |
| 9.Tasselli *et al*. (2014) | - How do multiple connections between single professionals (e.g., professionals working in the same organization, or members of the same scientific committee) entail connections between social groups and organizations? - How do the micro-level of interpersonal interactions and the macro-level involving connections between organizations coevolve? - Are dynamics in the structure of professionals’ networks affected by organizational change? Or does organizational change affect those dynamics? - How do dynamics in professionals’ networks shape dynamics in macro-level organizational outcomes, including strategic change and performance? |  | - We envisage the need for research on the bridge between micro-level, interpersonal networks and macro-level, interorganizational networks in predicting network outcomes. We also call for further research on network dynamics, aimed at investigating how social networks change over time and, through this change, affect the fates of health care organizations. |
| 10. Cunningham *et al*. (2012) | - Although a third of the studies link network structure with evidence of outcomes, most of the research examines only the structural features of health professional networks. |  | - Using multi-method approaches, and exploiting advances in SNA, further well designed research should examine the relationships between professionals' network structures and health outcomes in a range of different care settings, and how the structural aspects of health professional networks can be leveraged to improve quality of care and patient outcomes. - First, understanding the structure and characteristics of professional networks is vital, and second, it is important to attend to how they function. This leads to a third lesson: it may be time well spent, depending on local conditions, to nurture professional networks, and invest the time to facilitate their contributions to care. |
| 11.Chambers *et al*. (2012) | - What is striking is that nearly all the literature is descriptive in nature; and only one study has used the results of an SNA to bring about change, specifically to increase the use of personal order sets by physicians in a hospital information system. The major limitation of the review reflects the limitations of the evidence base and the almost complete lack of studies involving SNA as part of an intervention. - We found very little evidence for the potential of SNA being realised in healthcare settings. However, it seems unlikely that networks are less important in healthcare than other settings. |  | - Future research should seek to go beyond the merely descriptive to implement and evaluate SNA-based interventions. - There is currently an absence of evidence to demonstrate that using SNA can enable intelligent targeting of key relationships and collaborations to facilitate better uptake and utilisation of knowledge. Future studies involving SNA in healthcare should be designed with an intervention and comparator. SNAs can be either dependent or independent variables not divorced from any other intervention (independent variable) or measurement (dependent variable). There is a risk that SNA discourse and time may foster a separation from classical literature on attribution of change to causes and questions of bias; to avoid this, level I studies need to be adequately powered and designed with appropriate comparators. |
| 12. Dunn et al. (2011) | - The lack of validation in published healthcare social network analyses is a potential pit fall that may lead to misinterpretation as a result of confounding factors. This has the potential to become a serious impediment to the perceived quality of research using social network analysis in health care, and for the subsequent development of policy. |  | - We have presented a simple method for establishing a baseline from which a network analyst can validate the network metric values of individual small networks and then compare across networks with different sizes and densities. Armed with the knowledge of confounders and a method for validation, researchers and readers are better equipped to accurately interpret communication patterns and can more effectively compare their results within and between case studies. We propose that future case studies involving social network analysis in healthcare organisations would benefit from the application of this method, in turn providing more trustworthy support for the reengineering of social structures, work processes and other organisational policy development. |
| 13.Braithwaite *et al*. (2010) |  |  | - In discussing the circumstances in which network disjunctures occur, and how and when, we need to remember the goal is to formulate new ways of improving health sector organisational communications, knowledge transmission and relationships across pre-existing divides. |

1. Chambers D, Wilson P, Thompson C, Harden M. Social network analysis in healthcare settings: a systematic scoping review. PLoS One. 2012;7(8):e41911. [↑](#footnote-ref-1)
2. Sabot K, Wickremasinghe D, Blanchet K, Avan B, Schellenberg J. Use of social network analysis methods to study professional advice and performance among healthcare providers: a systematic review. Syst Rev. 2017;6(1):208. [↑](#footnote-ref-2)
3. Holly C, Salmond S, Saimbert M. Comprehensive systematic review for advanced nursing practice New York: Springer. 2012. p. 86. [↑](#footnote-ref-3)
4. Hannes K. Chapter 4: Critical appraisal of qualitative research In: Noyes J, Booth A, Hannes K, et al, eds Supplementary Guidance for Inclusion of Qualitative Research in Cochrane Systematic Reviews of Interventions Version 1 (updated Aug 2011). Cochrane Collaboration Qualitative Methods Group, 2011. <http://cqrmg.cochrane.org/supplemental-handbook-guidance>. [↑](#footnote-ref-4)
