# Supplementary material for: Review of social networks of professionals in healthcare settings—where are we and what else is needed?
Source: Global Health. 2021 Dec 4;17:139. doi: 10.1186/s12992-021-00772-7 (PMC8642762; doi:10.1186/s12992-021-00772-7)
Supplement: Supplementary file 4 — Appendix 12 PRISMA-Checklist. [file 12992_2021_772_MOESM4_ESM.doc]

## Appendix 14: Gaps and Suggestions about SNA regarding Healthcare Providers

| **Review** | **Gaps** |  | **Suggestions** |
| --- | --- | --- | --- |
| 1. Glegg et al. (2019) | - With more than half of the included studies examining physician-only networks, and only a handful studying interprofessional health care teams, great opportunity exists to expand the range of professions under study. Because of the growing shift in health services delivery from profession-based to collaborative practice models involving interprofessional teams [57], further research is needed to evaluate the generalizability of findings beyond physician networks, as well as in other health care contexts. - Because of its examination of information flow, predominantly, this body of research presents a narrow view of KT that focuses primarily at the individual level of evidence-based decision-making. This limitation relates in part to the scope of the review, as well as the consideration that other actors (e.g., health leaders, researchers) rather than health professionals may typically manage many of the KT activities that were not represented. |  | - The included articles focused on individual level evidence-based decision-making: we recommend also applying SNA to meso- or macro-level KT activities. SNA research that expands the range of professions under study, examines network dynamics over time, extends the depth of analysis of the role of network structure on KT processes and outcomes, and employs mixed methods to triangulate findings, is needed to advance the field. - Longitudinal research, a more representative range of populations, the use of interviews, document review and observation for data collection, greater depth of analysis, and the leveraging of network visualizations can augment the contributions of SNA to the KT science knowledge base. - Understanding how network properties can be used as proxies to measure social processes (e.g., information exchange, best practice adoption, decision-making, influence) can help KT scientists to apply SNA effectively to expand the range of measures that can be used to evaluate KT efforts. |
| 2. DuGoff et al. (2018) | - One gap centers on the actors used to construct the networks. Studies of providers focused on physicians and hospitals; only two studies included allied health professionals such as nurses and pharmacists. Given the importance of post-acute care in geographic variations in health care utilization, further work is needed to understand the interplay between post-acute care facilities and community-based physicians - Another area for further research is how patient-sharing networks change over time or in response to different incentives. Longitudinal studies may provide insight into the impact of policy reforms that seek to alter the ways in which patients receive care. - Finally, social network approaches are increasingly being deployed in public health initiatives [6, 68]. However, we did not identify studies that used patient-sharing networks to develop or evaluate an intervention. |  | - First, researchers should determine how many shared patients are necessary to form a tie between two nodes. - Second, researchers must determine which providers should be included or excluded. - Third, appropriate geographic boundaries need to be set for determining a provider network, which may influence the network’s structure. - Fourth, selecting an approach for identifying communities or clusters of providers may be challenging. We found Girvan-Newman’s algorithm to be the most popular, but other approaches are likely valid. |
| 3. Brunson et al. (2018) | - The network conceptual model is most illustratively called into question in the clinical co-occurrence setting: There are widely recognized problems with collapsing co-occurrence data to unipartite network models, but few studies of the “diseaseome” addressed them. - Several additional studies discussed biases in their data and tested the sensitivity of their results to different sources and thresholds, but these efforts were far narrower than the breadth of methods used, and none discussed concomitant differences in the resulting network structure. |  | - Some studies employed network measures without specific motivation, eg, degree and betweenness centrality as possible social determinants of methadone treatment continuation. In this case, a discernible effect of degree was given a reasonable interpretation, but an indiscernible effect of betweenness was not commented upon; had it been theoretically motivated, an account of this result would clearly be required. |
| 4. Sabot et al. (2017) | - Network methods are underutilized for the purposes of understanding professional communication and performance among healthcare providers. The paucity of articles meeting our search criteria, lack of studies in middle- and low-income contexts, limited number in non-tertiary settings, and few longitudinal, experimental designs, or network interventions present clear research gaps. - A general challenge generated by SNA methods is the need to clearly define the study boundary, which can limit sample size and therefore affect the broader generalizability. This came up in several studies noting areas for further research including broadening to other settings and repeating the study elsewhere given the limited sample size. The sample size limitation related less to the number of nodes, but more to the number and type of whole networks included. - The lack of longitudinal and experimental designs speaks to a broader challenge in the field as these are new areas for application of SNA methods, and the analytical tools and software are still in development. This limited the ability of SNA studies to address causal pathways. - Similarly, the limited qualitative methods being integrated into the studies constrain the contextual understanding of the network properties quantified and visualized through applying the quantitative SNA methods. |  | - One of the challenges facing researchers wanting to use SNA methods is the lack of validated SNA survey tools for use in the health sector, as highlighted by Creswick and Westbrook and Perkins et al. While this only is relevant for those interested in using sociometric survey methods, as more studies use SNA methods, we can anticipate that a set of tools or best practices for applying a range of SNA methods will emerge. |
| 5. Poghosyan et al. (2016) | - Team member demographic characteristics, professional affiliations, and clinical circumstances of patient populations can influence network formation. The findings were mixed regarding the influence of demographic characteristics. Some studies indicated that similarity in terms of age and gender may promote formation of the network while others did not find significant associations between these characteristics and network formation. - The review revealed many SNA studies conducted in other fields (e.g. sociology, management, engineering, etc.), the literature investigating health care team networks appears to be advancing slowly. |  | - None of the reviewed studies looked at the network dynamics over time. Network structures can be transient and will evolve over time as teams experience turnover, although such observations have yet to be tested longitudinally. Future research could focus on producing evidence about these changes to provide a deeper understanding about how networks regroup or fall apart and how their evolution affects patient care and outcomes. |
| 6. Mitchell *et al*. (2016) | - The review has highlighted that the adoption of an SNA to facilitate the improvement of care to residents in LTC has not yet made the same progress as in other health and clinical settings, where studies have linked network structure to evidence of outcomes. |  | - Further research into network structures could investigate how social networks improve staff's workplace experience, enable better care for residents, and contribute to organizational success in a constantly changing environment. |
| 7. Bae *et al*. (2015) | - Current studies on the social ties of health care workforce professionals include several assessments of inefficiencies. The level of technical sophistication in these studies tended to be low. - There is considerable risk for bias in the 28 included studies. There were no studies that used an intervention to attempt to change the structure or function of the social network and no designs that used experimental study design with a control group. |  | - Future study using enhanced sophistication in study design, analysis, and patient outcome testing are warranted to fully leverage the potential of SNA in health care studies. - To implement the interventions, researchers may need to change the current layout in health care settings and may have to use a longitudinal study design. SNA studies that involve altering the physical architecture of a unit present unique difficulty in finding appropriate study sites. |
| 8. Benton *et al*. (2015) | - So far it would appear that no author or group of authors have developed a programme of research in the nursing field using the social network analysis approach - The dominance of literature from North America may be viewed as problematic as the underlying structures and themes may be an artefact of cultural communication norms from this region. - The most significant limitation of the work conducted and published in the literature to this point relates to the one-off nature, single point-in-time and often small sample sizes exhibited by many of the studies. |  | - The lack of longitudinal studies and the absence of replication across multiple sites should be seen as an opportunity for further research. - In terms of how this technique might further contribute to our understanding of interventions on such factors as team communication or on the consequences of various learning interventions, more sophisticated designs would be required. However, the current dearth of multicentre and time-series designs could be viewed as a potential opportunity for further work. - There is a need to conduct more intervention-based studies where the underlying network structure is explored to gain insights into the outcomes of differing structures as well as the contribution that different actors play and how various intervention impact on network outcomes. |
| 9.Tasselli *et al*. (2014) | - How do multiple connections between single professionals (e.g., professionals working in the same organization, or members of the same scientific committee) entail connections between social groups and organizations? - How do the micro-level of interpersonal interactions and the macro-level involving connections between organizations coevolve? - Are dynamics in the structure of professionals’ networks affected by organizational change? Or does organizational change affect those dynamics? - How do dynamics in professionals’ networks shape dynamics in macro-level organizational outcomes, including strategic change and performance? |  | - We envisage the need for research on the bridge between micro-level, interpersonal networks and macro-level, interorganizational networks in predicting network outcomes. We also call for further research on network dynamics, aimed at investigating how social networks change over time and, through this change, affect the fates of health care organizations. |
| 10. Cunningham *et al*. (2012) | - Although a third of the studies link network structure with evidence of outcomes, most of the research examines only the structural features of health professional networks. |  | - Using multi-method approaches, and exploiting advances in SNA, further well designed research should examine the relationships between professionals' network structures and health outcomes in a range of different care settings, and how the structural aspects of health professional networks can be leveraged to improve quality of care and patient outcomes. - First, understanding the structure and characteristics of professional networks is vital, and second, it is important to attend to how they function. This leads to a third lesson: it may be time well spent, depending on local conditions, to nurture professional networks, and invest the time to facilitate their contributions to care. |
| 11.Chambers *et al*. (2012) | - What is striking is that nearly all the literature is descriptive in nature; and only one study has used the results of an SNA to bring about change, specifically to increase the use of personal order sets by physicians in a hospital information system. The major limitation of the review reflects the limitations of the evidence base and the almost complete lack of studies involving SNA as part of an intervention. - We found very little evidence for the potential of SNA being realised in healthcare settings. However, it seems unlikely that networks are less important in healthcare than other settings. |  | - Future research should seek to go beyond the merely descriptive to implement and evaluate SNA-based interventions. - There is currently an absence of evidence to demonstrate that using SNA can enable intelligent targeting of key relationships and collaborations to facilitate better uptake and utilisation of knowledge. Future studies involving SNA in healthcare should be designed with an intervention and comparator. SNAs can be either dependent or independent variables not divorced from any other intervention (independent variable) or measurement (dependent variable). There is a risk that SNA discourse and time may foster a separation from classical literature on attribution of change to causes and questions of bias; to avoid this, level I studies need to be adequately powered and designed with appropriate comparators. |
| 12. Dunn et al. (2011) | - The lack of validation in published healthcare social network analyses is a potential pit fall that may lead to misinterpretation as a result of confounding factors. This has the potential to become a serious impediment to the perceived quality of research using social network analysis in health care, and for the subsequent development of policy. |  | - We have presented a simple method for establishing a baseline from which a network analyst can validate the network metric values of individual small networks and then compare across networks with different sizes and densities. Armed with the knowledge of confounders and a method for validation, researchers and readers are better equipped to accurately interpret communication patterns and can more effectively compare their results within and between case studies. We propose that future case studies involving social network analysis in healthcare organisations would benefit from the application of this method, in turn providing more trustworthy support for the reengineering of social structures, work processes and other organisational policy development. |
| 13.Braithwaite *et al*. (2010) |  |  | - In discussing the circumstances in which network disjunctures occur, and how and when, we need to remember the goal is to formulate new ways of improving health sector organisational communications, knowledge transmission and relationships across pre-existing divides. |
